# A meta-analysis reveals the environmental and host factors shaping the structure and function of the shrimp microbiota

**DOI:** 10.7717/peerj.5382

**Published:** 2018-08-10

**Authors:** Fernanda Cornejo-Granados, Luigui Gallardo-Becerra, Miriam Leonardo-Reza, Juan Pablo Ochoa-Romo, Adrian Ochoa-Leyva

**Affiliations:** Departamento de Microbiología Molecular, Universidad Nacional Autónoma de México, Instituto de Biotecnología, Cuernavaca, Morelos, Mexico

**Keywords:** 16S rRNA, Shrimp microbiota, Meta-analysis, Metagenomics, High-throughput sequencing, PICRUST, Shrimp microbiome

## Abstract

The shrimp or prawn is the most valuable traded marine product in the world market today and its microbiota plays an essential role in its development, physiology, and health. The technological advances and dropping costs of high-throughput sequencing have increased the number of studies characterizing the shrimp microbiota. However, the application of different experimental and bioinformatics protocols makes it difficult to compare different studies to reach general conclusions about shrimp microbiota. To meet this necessity, we report the first meta-analysis of the microbiota from freshwater and marine shrimps using all publically available sequences of the 16S ribosomal gene (16S rRNA gene). We obtained data for 199 samples, in which 63.3% were from marine (*Alvinocaris longirostris*, *Litopenaeus vannamei* and *Penaeus monodon*), and 36.7% were from freshwater (*Macrobrachium asperulum, Macrobrachium nipponense, Macrobranchium rosenbergii, Neocaridina denticulata*) shrimps. Technical variations among studies, such as selected primers, hypervariable region, and sequencing platform showed a significant impact on the microbiota structure. Additionally, the ANOSIM and PERMANOVA analyses revealed that the most important biological factor in structuring the shrimp microbiota was the marine and freshwater environment (ANOSIM *R* = 0.54, *P* = 0.001; PERMANOVA pseudo-*F* = 21.8, *P* = 0.001), where freshwater showed higher bacterial diversity than marine shrimps. Then, for marine shrimps, the most relevant biological factors impacting the microbiota composition were lifestyle (ANOSIM *R* = 0.341, *P* = 0.001; PERMANOVA pseudo-*F* = 8.50, *P* = 0.0001), organ (ANOSIM *R* = 0.279, *P* = 0.001; PERMANOVA pseudo-*F* = 6.68, *P* = 0.001) and developmental stage (ANOSIM *R* = 0.240, *P* = 0.001; PERMANOVA pseudo-*F* = 5.05, *P* = 0.001). According to the lifestyle, organ, developmental stage, diet, and health status, the highest diversity were for wild-type, intestine, adult, wild-type diet, and healthy samples, respectively. Additionally, we used PICRUSt to predict the potential functions of the microbiota, and we found that the organ had more differentially enriched functions (93), followed by developmental stage (12) and lifestyle (9). Our analysis demonstrated that despite the impact of technical and bioinformatics factors, the biological factors were also statistically significant in shaping the microbiota. These results show that cross-study comparisons are a valuable resource for the improvement of the shrimp microbiota and microbiome fields. Thus, it is important that future studies make public their sequencing data, allowing other researchers to reach more powerful conclusions about the microbiota in this non-model organism. To our knowledge, this is the first meta-analysis that aims to define the shrimp microbiota.

## Introduction

The microbiota plays essential roles in the development and physiology of their host, such as preventing the growth of pathogenic bacteria, modulating the immune response, nutrient absorption, regulating metabolic processes and producing vitamins (Bikel et al., 2015). The surrounding environment of water and sediment also plays a significant role in modulating the microbiota composition of animals from aquatic systems such as crustaceans, including crabs (Zhang et al., 2017), lobsters (Feinman et al., 2017), and shrimps (Cornejo-Granados et al., 2017). The shrimp or prawn is the most valuable traded marine product in the world today. The earliest study on shrimp microbiota dates from 1961, in which bacterial communities were isolated from shrimp organs using traditional microbiology approaches (Tysset, Mailloux & Brisou, 1961). Afterward, techniques such as denaturing gradient gel electrophoresis (DGGE) and clone libraries (Stackebrandt, Liesack & Goebel, 1993) also began to be used to characterize shrimp bacterial communities. The recent advances in high-throughput sequencing of the small ribosome subunit 16S gene (16S rRNA gene), plus the importance of this organism for commercial distribution, have increased the interest in characterizing the bacterial communities of shrimps and its habitat (Durand et al., 2010; Rungrassamee et al., 2014; Mente et al., 2016; Xiong et al., 2017a; Cornejo-Granados et al., 2017; Cui et al., 2017). So far the effects that health status, developmental stage and diet have on shrimp microbiota have been studied under laboratory and pond-reared aquaculture hatchery conditions (Mente et al., 2016; Zeng et al., 2017; Xiong et al., 2017b). However, all the studies mentioned above used different experimental and bioinformatics protocols making it difficult to compare the results between studies.

There are significant technical and bioinformatics biases when comparing microbiota results of different studies. The technical differences mainly include the selection of the amplified 16S rRNA hypervariable region, the use of different PCR primers for the same hypervariable region and DNA extraction protocols; while bioinformatics differences include the database selection for taxonomy assignment, the use of different clustering algorithms, and the quality filtering of sequences (Bikel et al., 2015). The impact of these factors on the microbiota diversity has been discussed in other meta-analyses (De Filippis et al., 2018; Lozupone et al., 2013). These biases can be minimized for 16S rRNA amplicon studies using public data and analyzing them with similar bioinformatics methods, helping to establish the best protocols to characterize the microbiota of a given niche. To meet these research needs, we present a meta-analysis of shrimp microbiota using all available high-throughput 16S rRNA sequencing data. The meta-analysis was conducted using the same bioinformatics protocol allowing us to explore the impact that biological factors such as habitat, farm, laboratory, organ, developmental stage, disease, and diet, have on the microbiota structure and composition, after the known biases introduced by experimental and technical issues of each study. To our knowledge, this is the first meta-analysis that aims to define the shrimp microbiota based on all publicly available data of 16S rRNA amplicon sequencing.

## Materials & Methods

### Identification of relevant studies and data collection

To develop this study we systematically reviewed all available studies related to shrimp or prawn microbiota. The relevant studies were identified by systematic searches of the SCOPUS database using 37 keywords on February 1 of 2018 (Table S1). This search resulted in 536 articles from which the title and abstracts were screened by all the authors (FCG, LGB, MLR, JPOR, and AOL) for their suitability for this meta-analysis. We excluded two main types of studies: (1) books, reviews, meta-analysis studies, conference papers, and theses; (2) studies in organisms different from shrimp species. After that, we obtained 110 studies, which were grouped into those that used culture-dependent or culture-independent techniques for bacterial community characterization. To conduct the meta-analysis the studies also had to include the following: (i) freely available 16S rRNA sequencing data; and (ii) sequencing data correctly separated by sample type. Any disagreement was carefully discussed among the five authors to reach a final decision. This process led to 16 studies that grouped 199 samples (Table 1 and Table S2). We obtained the sequencing reads from GenBank and DDBJ. The Prisma flow diagram depicting the search protocol and workflow of our meta-analysis is in Fig. S1.

**Table 1:** Articles with publicly available sequencing data used for shrimp microbiota meta-analysis (16 articles).

**Reference**	**Hypervariable region**	**Primers**	**Sequencing technology**	**Shrimp specie**	**No. of samples**	**Sample type(s)**	**Country**	**Accession number**
Zheng et al. (2017)	V3–V6	341F 1073R	Roche 454	*Litopenaeus vannamei*	17	Whole larvae and post larvae	China	SRP080243
Chen et al. (2017a); Chen et al. (2017b)	V1–V2	27F 355R	Illumina MiSeq	*Macrobrachium nipponense*	19	Intestine	Taiwan	SRP094102
Suo et al. (2017)	V4–V5	515F 907R	Illumina MiSeq	*Litopenaeus vannamei*	15	Intestine	China	SRP091598
Rungrassamee et al. (2014)	V3–V4	338F 518R	Roche 454	*Penaeus monodon*	6	Clean intestine	Thailand	KF329429 –KF334451, KF334452 –KF344403, KF344404 –KF355928, KF322280 –KF325238, KF325239 –KF328420, KF328421 –KF329428
Cornejo-Granados et al. (2017)	V2, V3, V4, V5, V6–7, V8, V9	Ion 16S™ Metagenomics Kit	Ion Torrent	*Litopenaeus vannamei*	18	Intestine and hepatopancreas	Mexico	SRP107821
Gainza et al. (2017)	V2–V3	341F 518R	Ion torrent	*Litopenaeus vannamei*	14	Intestine	Ecuador	SRP092753
Qiao et al. (2017)	V4–V5	515F 907R	Illumina MiSeq	*Litopenaeus vannamei*	9	Intestine	China	SRP061605
Sun et al. (2016)	V3–V4	341F 806R	Illumina MiSeq	*Alvinocaris longirostris*	2	Gill and intesitine	Japan	SRP064953
Tzeng et al. (2015)	V1–V2	27F 355R	Roche 454	*Macrobrachium asperulum, Macrobrachium nipponense*	6	Intestine	Taiwan	SRP057429
Oetama et al. (2016)	V4	515F 806R	Illumina MiSeq	*Penaeus monodon*	3	Stool	Indonesia	SRP059721
Zhang et al. (2014a); Zhang et al. (2014b)	V4–V5	515F 907R	Illumina MiSeq	*Litopenaeus vannamei*	18	Intestine	China	SRP043399
Zhang et al. (2016)	V4–V5	515F 907R	Illumina MiSeq	*Litopenaeus vannamei*	8	Intestine	China	SRP051489
Rungrassamee et al. (2013)	V3–V6	338F 786R	Roche 454	*Penaeus monodon*	4	Intestine	Thailand	JX919344 –JX926388, JX916289 –JX919343, JX926389 –JX939518, JX939519 –JX941408.
Rungrassamee et al. (2016)	V3–V4	338F 518R	Roche 454	* Penaeus monodon*	12	Intestine	Thailand	KP944208 –KP944681, KP948364 –KP948529, KP944682 –KP946571, KP946572 –KP946691, KP946692 –KP948363, KP948530 –KP948831, KP948832 –KP951735, KP953299 –KP953763, KP951736 –KP952247, KP952248 –KP952978, KP952979 –KP953298, KP953764 –KP953903
Cheung et al. (2015)	V1–V3	28F 519R	Ion torrent	*Neocardina denticulata*	27	Foregut, intestine, hepatopancreas	China	SRR1735538
Mente et al. (2016)	V3–V4	S-DBact-0341-b-S-17 S-D-Bact-0785-a-A-21	Roche 454	*Macrobrachium rosenbergii*	21	Clean intestine	USA	SRR1502207

### Meta-analysis

To keep most of the samples across all analyses, we decided to filter all sequences maintaining a minimum quality of Q20, a minimum length of 90 bp and discarding all sequences with ambiguous nucleotides. The remaining sequences (17,515,413) partitioned by sample, were clustered at 97% identity into operational taxonomic units (OTUs) against the Greengenes database (version 13_8) using UCLUST in QIIME 1.9.1(Caporaso et al., 2010). The reverse strand matching option was enabled, and we discarded the reads that failed to match a reference sequence for downstream analyses. We directly assigned the taxonomy from the Greengenes database based on the identity with the reference sequence clustered. We selected the closed-reference OTU picking command because we were comparing non-overlapping amplicons. This OTU picking method is a reference-based approach; thus, chimera removal was not necessary. After that, we assigned the taxonomy for a total of 10,596,387 high-quality filtered reads, with a 277 bp mean read length for 199 samples. We eliminated the OTUs represented by a single read (singleton) or with a frequency ≤0.005 for further analyses, which helped to keep the estimates of *α*-diversity realistic and to avoid information loss. Taxonomy summaries with relative abundance data were subsequently generated and averaged. The most abundant sequence within an OTU was selected as the OTU’s representative, and these representative sequences were then aligned against Green Genes using the align_seqs.py command and PyNAST with a minimum sequence identity of 75%. The alignment was filtered using filer_alignment.py, and a phylogenic tree was constructed using the make_phylogeny.py command with the FastTree method for tree building. Alpha and beta diversity metrics from the final OTU table without singletons were obtained using the QIIME 1.9.1(Caporaso et al., 2010). To increase the sequence depth for alpha and beta diversity analysis, we discarded 31 samples with low sequencing depth. Thus, the alpha diversity metrics (Observed OTUs, Shannon, and Phylogenetic Diversity PD) were calculated at a sequence depth of 1,108 reads per sample with 10,000 iterations and then were averaged. The selected maximum sampling depth corresponded to the minimum number of reads obtained for any of the remaining sequenced samples. Beta diversity was estimated by computing from the phylogenetic tree the unweighted UniFrac distances among samples at a sequence depth of 1,108 reads per sample, and the UniFrac distance matrices were visualized using principal coordinates analysis (PCoA) in QIIME with the beta_diversity_through_plots.py command. The plots were made using the R package ggplot2 (Wickham, 2016), and the ellipses represented the normal distribution with a confidence level = 0.95 for each group. To explore the most abundant bacterial taxa in the PCoA space we produced the biplots using QIIME 1.9.1, where the abundance of bacterial taxa was plotted in the same PCoA space based on the average of weighted abundance for all samples, this refers to the relative abundance of the taxon in the samples (Lozupone et al., 2013). A permutational multivariate analysis of variance (PERMANOVA) (Anderson, 2014) with Adonis function on the unweighted UniFrac and Bray-Curtis distance matrices was used within QIIME to quantitatively evaluate the effects that the different habitat and host factors (organ, diet, lifestyle, and developmental stage) had on shrimp microbiota. Also, we evaluated the effects of technical factors such as primers used for amplification, study, hypervariable regions, country, and sequencer. The analysis of similarities (ANOSIM) on the unweighted UniFrac distances between factors was conducted in QIIME. The final OTU table from marine samples was also used as an input for functional metagenomic prediction using PICRUSt. The KEGG pathway content obtained by PICRUSt was normalized and then used to obtain the metagenomic functional predictions at different hierarchical KEGG levels (1, 2 and 3). To determine the taxonomic classifications and predicted functions that were significantly more abundant in each group of samples we applied a Wilcoxon’s non-parametric rank-sum test, followed by LDA using the LEfSe program (Segata et al., 2011).

### Accession number

The accession numbers of the reads used in this meta-analysis are in Table S2.

## Results

### Systematic search results

**Figure 1:**
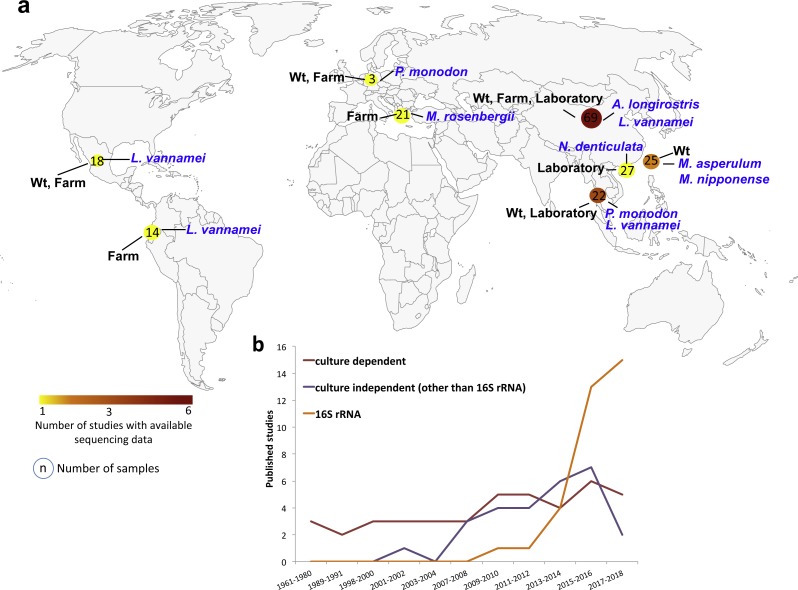
Geographic and year distribution of studies about shrimp microbiota. (A) Geographic distribution of all studies with publically available sequencing data (Table 1). The shrimp species, lifestyle condition, and the number of sequenced samples are show for the countries. (B) Year distribution of all studies grouped into the use of culture-dependent, culture-independent or 16S rRNA gene sequencing.

The bacterial communities of shrimp organs have been earlier studied using traditional approaches mainly based on cultivable bacteria (Fig. 1). The advances on molecular biology and cultivation techniques led to an increase in molecular studies in the first decade of the 2000s, mainly due to the application of denaturing gradient gel electrophoresis (DGGE) and clone libraries (Fig. 1). Interestingly, the dropping costs of high-throughput sequencing of the small ribosome subunit 16S gene (16S rRNA gene) facilitated a faster growth of microbiota studies in the last decade (Fig. 1). We found 30 studies using high-throughput sequencing of the 16SrRNA gene to characterize the shrimp microbiota (Table S3), 19 of them have freely available sequencing data in public repositories. However, only 16 studies provided the reads adequately identified and separated in different files for each sample. Thus, for our meta-analysis, we only focused on the sequencing data from these 16 studies (Table 1). The retrieved data corresponded to PCR-amplicons targeting different hypervariable regions of the bacterial 16S rRNA gene and they were sequenced using different sequencing platforms (Table 1).

The studies collected 199 samples from China (47.2%), Taiwan (12.6%), Thailand (11.1%), USA (10.6%), Mexico (9.0%), Ecuador (7.0%), Indonesia (1.5%) and Japan (1.0%) (Fig. 1, Table S2). A total of 63.3% of the samples came from marine (*Alvinocaris longirostris, Litopenaeus vannamei and Penaeus monodon*), and 36.7% came from freshwater (*Macrobrachium asperulum, Macrobrachium nipponense, Macrobranchium rosenbergii, Neocaridina denticulata*) shrimps (Table S2). 48.2% of the samples were from laboratories, 32.7% from farms and 19.1% from wild-type (wt) lifestyles. The 75.9% of the samples corresponded to the intestine, 9.0% to hepatopancreas, 8.5% to whole shrimp, 1.5% to shrimp stool and 0.5% to the gill. The samples represented different developmental stages, adult (45.2%), juvenile (41.2%), larvae (11.6%) and post-larvae (2.0%). A total of 88.9% samples were from healthy and 11.1% from diseased shrimps. In total, we analyzed seven shrimp species where 52.8% of samples were from *L. vannamei*, 13.6% from *N*. denticulate, 12.1% from *M. nipponense*, 10.6% from *M. rosenbergii*, 9.5% from *P. monodon*, 1.0% from *A. longirostris* and 0.5% from *M. asperulum* (Table S2). Interestingly, gathering all studies, the nine hypervariable regions of the 16S RNA gene have been sequenced, V4–V5 being the most used region. 37.2% of the samples were sequenced using Illumina MiSeq, 33.2% Roche 454 and 29.6% Ion Torrent sequencing technologies (Table S2).

### Meta-analysis

#### General microbiota characteristics

The dominant phylum present in all the samples was Proteobacteria (average = 65.99%) (Fig. S2a), revealing that most bacteria species from the intestine, hepatopancreas, stool, gill and whole shrimp are from this phylum. In addition, members of Firmicutes (average = 16.42%), Actinobacteria (average = 3.24%), Bacteroidetes (average = 2.17%), and Fusobacteria (average = 0.76%) accounted for the 88% of total sequences (Fig. S2a). The alpha diversity indices were calculated using the rarefaction curves at OTUs level at a sequencing depth of 1,108 where Shannon, PD and observed OTUs indices were stable (Fig. S3). To reach this sequencing depth we removed 15 samples from this analysis. The Good’s coverage revealed that we obtained on average ∼99% ± 0.62% of the total OTUs for all the analyzed samples, indicating a good sequencing depth to represent the bacterial community. Interestingly, the freshwater (PD = 6.2 ± 3.5) samples had larger phylogenetic diversity than marine samples (PD = 4.9 ± 2.7).

#### The environment (marine and freshwater) drives the clustering and diversity of shrimp microbiota

**Figure 2:**
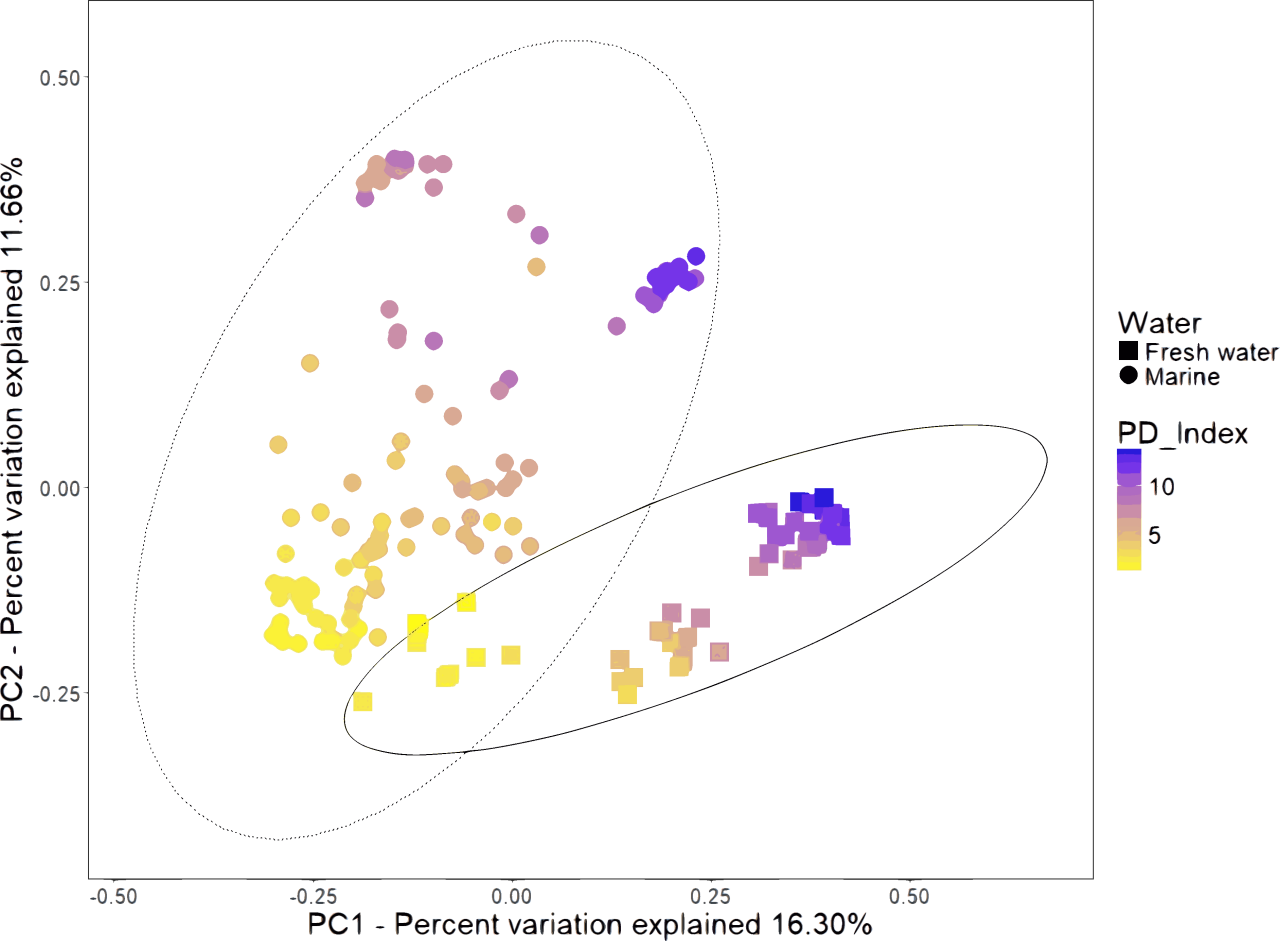
Beta diversity analysis of microbiota samples from freshwater and marine shrimps. Unweighted principal coordinate analysis (PCoA) of UniFrac distances for samples tagged by marine or freshwater origin. The color gradient shows the value of the Phylogenetic Diversity index (PD). The ellipses represented the normal distribution with a confidence level = 0.95 for each group.

We combined the sequencing data of 16 studies gathering 199 samples (Table S2). The PCoA of unweighted UniFrac distances revealed that samples formed several clusters (Fig. 2). A one-way analysis of similarity (ANOSIM) using the unweighted UniFrac distances revealed that the most significant separation is given by technical factors such as study (*R* = 0.984, *P* = 0.001), primers used for amplification (*R* = 0.846, *P* = 0.001) and hypervariable region (*R* = 0.817, *P* = 0.001). The fourth most important effect is given by marine and freshwater environment (*R* = 0.561; *P* = 0.001). We considered this the main biological factor that drives the shrimp microbiota. The most abundant bacterial orders were superimposed on the same PCoA plot (biplot), to know which orders were driving the diversity according to the freshwater or marine origin. We found that Enterobacteriales, Vibrionales, Rhodobacteriales and Alteromonadales orders drive clustering for marine shrimps, while Burkholderiales and Clostridiales drive clustering for freshwater shrimps (Fig. S4).

The bulk of OTU abundance at phylum level showed that Proteobacteria dominated the microbiota of marine samples (88.6%), as compared to freshwater samples (52.4%). Tenericutes and Fusobacteria were most abundant in marine samples with 2.0 and 1.8% respectively as compared to freshwater samples with 0.000077 and 0.4% (Fig. S2a). In contrast, Firmicutes was more abundant in freshwater (32.1%) versus marine samples (1.24%). The families Rhodobacteraceae (representing 3.1% of the total reads), Vibrionaceae (48.6%), Helicobacteraceae (2.2%) and Pseudoalteromonadaceae (18.7%) were the most abundant in marine samples, while the Oxalobacteraceae (10.8%), Comamonadaceae (6.8%), and Bacillaceae (4.1%) were the most enriched in freshwater samples (Fig. S2b). Although, it is important to note that samples from several shrimp species are too small to draw reliable conclusions.

**Figure 3:**
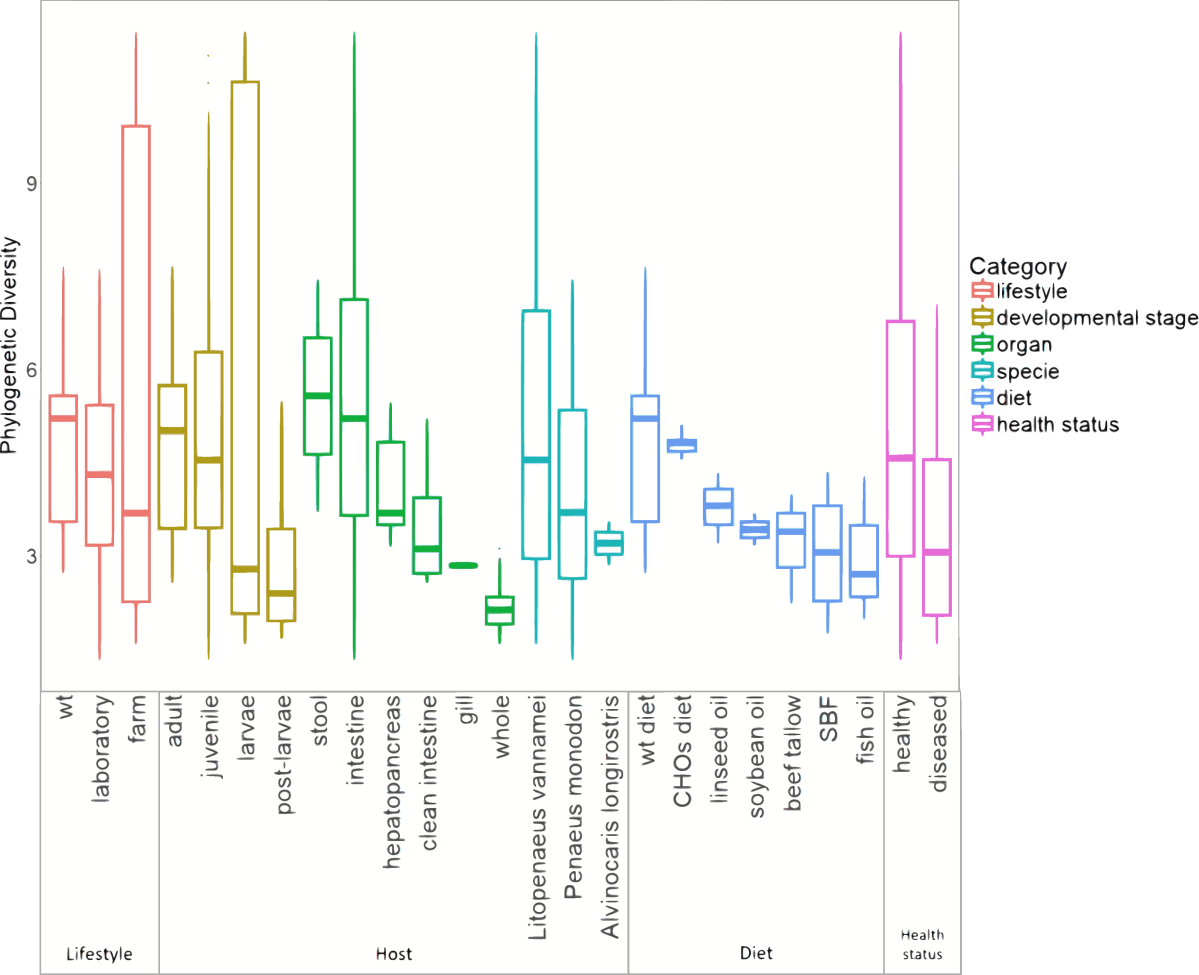
Alpha diversity of microbiota samples from marine shrimps. The Boxplots indicated the phylogenetic diversity index (PD) for all samples grouped by lifestyle, host, diet and health status categories. A sequence depth of 1,108 reads and 10,000 iterations were used to calculate the PD value.

The marine group of samples was the most abundant with 126 from the 199 samples, and it also includes the highest number of sample types, involving different lifestyles, developmental stages, and organs (Table S4). Furthermore, in the basis of the main separation observed in the PCoA between marine and freshwater samples (ANOSIM *R* = 0.561; *p* = 0.001), we performed all further analyses only considering the 126 marine samples. This allowed us to evaluate the impact that several biological factors such as lifestyle, developmental stages, and organs have on microbiota structure and function. For the alpha diversity analysis, all marine samples were further separated into six categories according to the conditions revealed in the original study: lifestyle, developmental stage, organ, species, diet, and health status (Fig. 3). The Shannon indices had similar diversity tendencies for all the categories (Fig. S5a). Concerning the lifestyle, the wild-type had more phylogenetic diversity than farm and laboratory samples (Fig. 3). In the developmental stage category, the highest diversity was for the adult, followed by the juvenile, larvae, and post-larvae in marine samples (Fig. 3). The stool samples showed higher phylogenetic diversity than shrimp organs, in which the top diversity was for intestine, followed by the hepatopancreas, clean intestine, gill and whole shrimp samples. Regarding the diet, we observed that wild-type diet increases the microbiota diversity while diets using different lipid sources decreases the microbiota diversity. Finally, as noted in previous studies (Xiong, Zhu & Zhang, 2014; Rungrassamee et al., 2016; Chen et al., 2017a; Zheng et al., 2017; Cornejo-Granados et al., 2017) samples from diseased shrimps showed lower diversity than samples from healthy shrimps.

#### Lifestyle conditions (wild-type, laboratory or farm) impact the clustering and diversity of marine shrimp microbiota

The analysis of similarities (ANOSIM) and permutational multivariate analysis of variance (PERMANOVA) of unweighted UniFrac distances revealed a significant association between the microbiota of the 126 marine samples and technical factors (Table 2). Additionally, grouping these samples by biological factors also was statistically significant (Table 2) such as lifestyle (ANOSIM *R* = 0.341, *P* = 0.001; PERMANOVA pseudo-*F* = 8.50, *P* = 0.0001), organ (ANOSIM *R* = 0.279, *P* = 0.001; PERMANOVA pseudo-*F* = 6.68, *P* = 0.001) and developmental stage (ANOSIM *R* = 0.240, *P* = 0.001; PERMANOVA pseudo-*F* = 5.05, *P* = 0.001). On the other hand, the diet was not considered in these analyses given that only 40 samples specified the ingredients of the diet. Bray-Curtis distances also showed similar contribution for all the factors described above (Table S5).

**Table 2:** Technical and biological factors associated with the microbial structure of shrimp microbiota. The impact was measured using Anosim (*R* value) and PERMANOVA with the adonis function (*F* and *R*_2_ values) of Unweighted UniFrac distances. For each analysis we performed 1,000 permutations to obtain the *p* value.

**Unweighted UniFrac**							
	**Parameter**	**ANOSIM**		**PERMANOVA**			
		***R***	***P* value**	**pseudo-*F***	***P* value**	*R*^**2**^	***P* value**
Technical factors	Paper	0.970	0.001	19.866	0.001	0.667	0.001
	Primer	0.663	0.001	12.346	0.001	0.459	0.001
	Hypervariable region	0.659	0.001	14.482	0.001	0.410	0.001
	Country	0.522	0.001	10.753	0.001	0.341	0.001
	Sequencer	0.493	0.001	16.097	0.001	0.231	0.001
Biological factors	Lifestyle	0.333	0.001	8.630	0.001	0.139	0.001
	Organ	0.261	0.001	6.998	0.001	0.252	0.001
	Developmental stage	0.215	0.001	5.075	0.001	0.126	0.001

**Figure 4:**
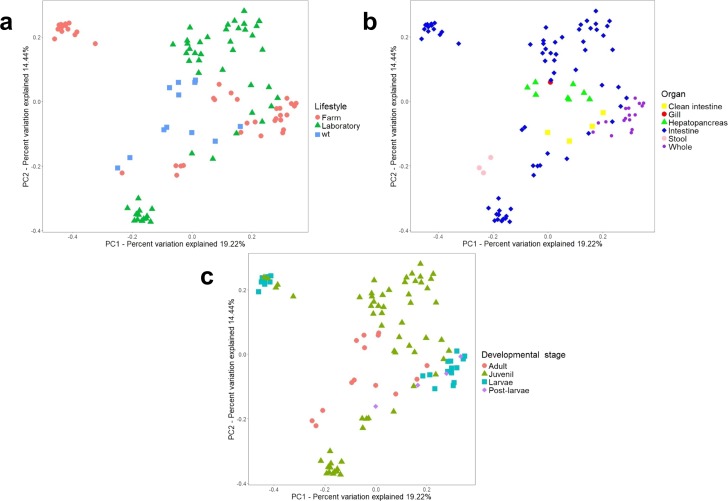
Beta diversity analysis of microbiota samples from marine shrimps. Unweighted principal coordinate analysis (PCoA) of UniFrac distances with samples tagged by (A) lifestyle, (B) organ and (C) developmental stage.

**Figure 5:**
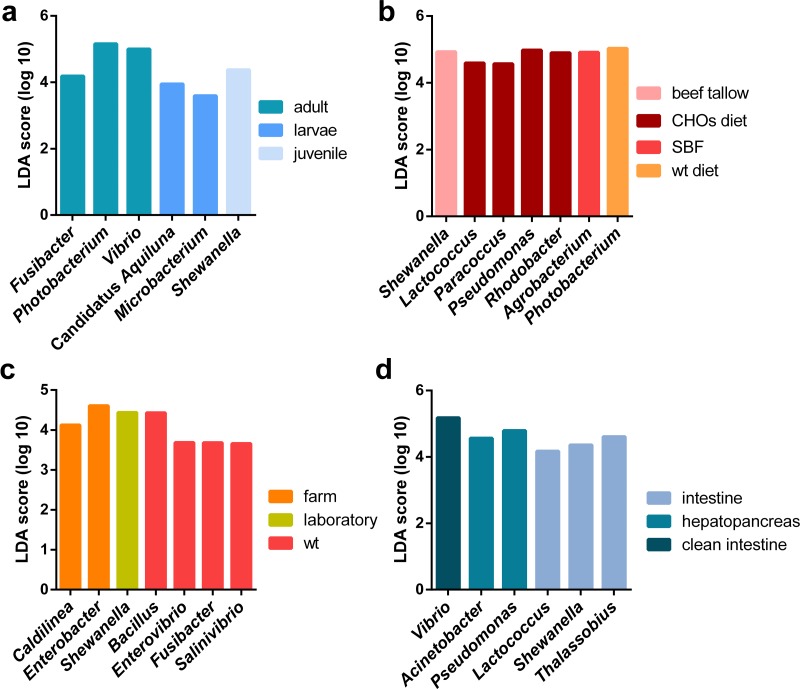
LEfSE results of enriched genera for all marine shrimp samples. All samples were analyzed to obtain the enriched genera in the following categories: (A) lifestyle, (B) organ, (C) developmental stage and (D) diet. The graph shows the log10 LDA score for each classification.

Additionally, a principal component analysis (PCoA) using unweighted UniFrac distances confirmed the organization in different clusters when we tagged the samples by biological factors such as lifestyle (Fig. 4A), organ (Fig. 4B), and developmental stage (Fig. 4C). When the most abundant bacterial orders were superimposed on the same PCoA plot (biplot), we observe that the clustering for wild-type, farm, and laboratory samples was driven by Vibrionaceae, Enterobacteriaceae, and Rhodobactereaceae respectively (Fig. S6a). The wild-type had most considerable phylogenetic diversity than farm and laboratory samples (Fig. 3). Genera that were enriched in specific lifestyles were identified using the linear discriminant analysis (LDA) effect size (LEfSe). Interestingly, 30 genera were differentially abundant between the three lifestyles (farm, laboratory and wt) (Fig. 5A).

#### Host factors (organ and developmental stage) impact the clustering and diversity of marine shrimp microbiota

Regarding the organ, the intestine has more phylogenetic diversity, followed by hepatopancreas, clean intestine (without fecal matter), gill and whole organism (Fig. 3). The intestine was the only organ where we observed members of the 19 phyla identified in the general taxonomy. The most abundant phyla for all the shrimp organs was Proteobacteria, with a more significant abundance in gill, followed by the whole organism, hepatopancreas, stool, clean intestine, and intestine (Fig. S5b). Tenericutes was found only in samples from the intestine, hepatopancreas, and stool. Helicobacteriaceae was the most abundant family in gill (81.2%), Vibrionaceae in the clean intestines (59.4%) and Enterobacteriaceae in whole shrimp (94.4%). Enterobacteriaceae (59.68%), Vibrionaceae (16.23%) and Pseudomonadaceae (11.42%) were the most abundant families in hepatopancreas. Finally, the abundance of families was more homogeneous in the intestine, but Mollicutes (10.71%), Rhodobacteraceae (11.56%) and Vibrionaceae (12.15%) were the most abundant (Fig. S2b). LDA analysis showed that the most enriched genera were Acinetobacter in the stool, Lactococcus in the intestine, Pseudomonas in the hepatopancreas and Vibrio in the clean intestine samples (Fig. 5B). These differences in the relative abundance of specific genera reflect the impact of the physiological conditions imposed by each organ.

When samples were tagged by developmental stage, we also observed clustering (Fig. 4C), that was confirmed by ANOSIM (Table 2). The Proteobacteria, Bacteroidetes, and Firmicutes were the most abundant phyla for all shrimp developmental stages (Fig. S2a). Although the two main phyla were present at all developmental stages, the most abundant bacterial groups shifted from one developmental stage to the other. For example, enrichment of Enterobacteriaceae was observed in larvae, followed by a progression to communities enriched in Rhodobacteraceae, Aeromonadaceae, and Mollicutes in juveniles and Vibrionaceae and Pseudomonadaceae in adults. The highest phylogenetic diversity was observed in the adult, followed by juvenile, larvae, and post-larvae (Fig. 3). The biplot showed that Vibrionaceae drove the adult cluster, while the juvenile was driven by Rodobactereaceae and an unidentified order from the Mollicutes class, and larvae and post-larvae by Enterobacteriaceae (Fig. S6b). The LDA analysis showed 17 differentially enriched genera according to the developmental stage (Fig. 5C).

Regarding the shrimp diet, we only analyzed the 40 samples that specified the diet ingredients in the original studies, leaving aside the impact of the different diet composition of commercial diets used in the other shrimp samples. The 40 samples were from seven diets: wild-type diet, diets supplemented with different carbohydrates (CHOs) sources (glucose, sucrose, and cornstarch) and diets supplemented with five different lipid sources (soybean oil, beef tallow, linseed oil, fish oil and SBF which is an equal combination of several lipid sources). Interestingly the wild-type diet was the one with the highest bacterial diversity, followed by the CHOs diet. In contrast, the diets using different lipid sources had the lowest bacterial diversity (Fig. 3), suggesting that diet imposes a selective pressure that shapes the bacterial community in shrimps. The LDA analysis showed seven differentially enriched genera according to the diet (Fig. 5B).

#### The disease has the lowest impact on bacterial clustering

After analyzing the samples in the PCoA tagged according to the health status, there was no effect in clustering due to disease (ANOSIM *R* = 0.025, *P* = 0.332) (Fig. S7). However, we observed that diseased shrimps had lower PD than healthy shrimps (Fig. 3). Regarding shrimp species, *L. vannamei* showed the most significant PD, followed by *P. monodon* and *A. longirostris*. However, the sample size could be influencing this result. Given that *L. vannamei* represented 83.3% of all marine samples, we analyzed them separately; these 105 samples represented five different developmental stages and three different organs. After this analysis, we also observed a similar clustering effect by lifestyle, organ, and developmental stage than observed in all the marine samples (Fig. S8).

### Functional potential of shrimp microbiota

It is known that in other organisms, taxonomic profiles are highly variable even among individuals (Lozupone et al., 2013); however, functions seem to remain stable (Human Microbiome Project Consortium, 2012) (functional redundancy). To further investigate the functional divergence among microbiomes of marine shrimps, we predicted the metagenome functions using PICRUSt (Langille et al., 2013). A total of 352 KEGG pathways were predicted for the 126 sequenced samples. Interestingly, the potential functions of the microbial community were significantly different among the biological factors (Fig. S9). LEfSE results indicated that several predicted pathways were significantly enriched among the samples. The most differentially enriched functions were among the organs (93 functions), followed by the different developmental stages (12 functions), and lifestyles (nine functions). The results suggested that the microbial functions varied a lot according to biological factors, being the organ the factor with more different functions.

## Discussion

Given the rising interest in the production of shrimp and the impact that microbiota has in shaping the health status and development of this organism, there is an increase in the number of studies that use high-throughput sequencing for the characterization of bacterial communities of the shrimp under different conditions. In this meta-analysis, we aimed to integrate all publically available data from high-throughput 16S rRNA gene sequencing studies using the same bioinformatics protocol minimizing the bias introduced by bioinformatics analysis, allowing us to establish which factors drive the structure and function of the shrimp microbiota. After assigning taxonomy for all the 199 samples, we observed a dominant presence of Proteobacteria, Firmicutes and Bacteroidetes (Fig. S2a), all of which have been previously reported to dominate the microbiota of shrimps and other aquatic organisms such as zebrafish (Roeselers et al., 2011) and salmon (Dehler, Secombes & Martin, 2017). Although all samples have different sequencing depth (2,000-1,000,000 reads), the rarefaction curves showed that most samples seem to reach saturation for the PD, Shannon and Observed OTUs metrics (Fig. S3), plus the mean Good’s Coverage value of ∼99% revealed that samples have good sequencing depth to represent the majority of the bacterial communities. Importantly, to not skew the observed clusters in the PCoA analysis towards the samples with the highest sequencing depth as previously reported (Lemos et al., 2011), we only analyzed the results of the unweighted UniFrac distances, which only consider the presence/absence of OTUs. First, we observed that the sequencing platform drives the clustering of samples, which is consistent with previous reports (Lozupone et al., 2013). Additionally, phylogenetic diversity showed that freshwater samples had higher diversity than marine samples, this is in agreement with a previous study that reports a higher bacterial richness in freshwater sediments than in marine (Wang et al., 2012). We found that the marine cluster was driven mainly by Burkholderiales order (29.2%) which was previously published as important in modulating the microbiota of shrimps from river and lake (Chen et al., 2017a).

**Figure 6:**
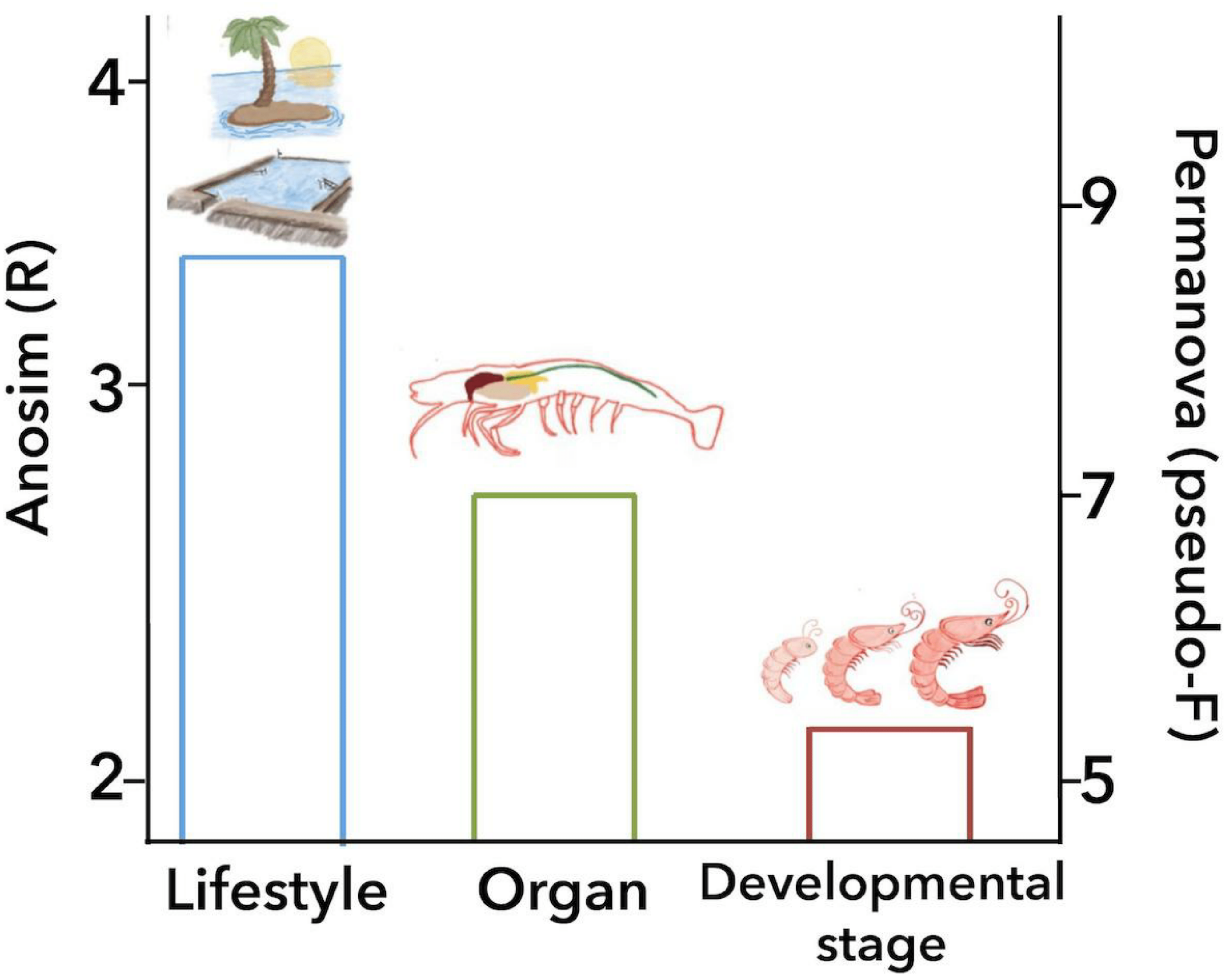
Principal biological factors that drive the microbiota variation in marine shrimps. The graph shows the ANOSIM *R* value (left axis) and the PERMANOVA pseudo-*F* value (right axis) obtained for the main biological factors that impact the shrimp microbiota: lifestyle, organ and developmental stage.

ANOSIM and PERMANOVA analyses confirmed that the technical factors have a great impact on the structure of the microbiota and that the strongest biological impact is given by freshwater or marine environment (Fig. 2). After that, we only selected the marine samples and confirmed that within this group, the main clustering effect is also due to technical and experimental factors used in each study (Table 2 and Fig. S10). This suggests that technical and experimental differences such as the primers, hypervariable regions of the 16S rRNA targeted, sequencing platforms, and DNA extraction methods cause significant differences in the microbiota, highlighting the importance of standardizing experimental and analysis protocols. A similar effect has been previously reported in other organisms such as swine (Holman et al., 2017), and humans (Lozupone et al., 2013). Aside from technical factors, the ANOSIM and PERMANOVA analyses showed that marine samples clustered significantly (Table 2) by lifestyle (ANOSIM *R* = 0.341, *P* = 0.001; PERMANOVA pseudo-*F* = 8.50, *P* = 0.0001), organ (ANOSIM *R* = 0.279, *P* = 0.001; PERMANOVA pseudo-*F* = 6.68, *P* = 0.001) and developmental stage (ANOSIM *R* = 0.240, *P* = 0.001; PERMANOVA pseudo-*F* = 5.05, *P* = 0.001) (Fig. 6). These results showed that environmental and host factors contribute significantly to shape the structure and composition of the shrimp microbiota independently of the technical factors.

After analyzing the samples by organ, we found that stool samples had the highest diversity, followed by the intestine, according to the PD index and by the hepatopancreas according to the Shannon index (Fig. 3 and Fig. S5a). This observation is consistent with the diversity indices previously reported (Cornejo-Granados et al., 2017). This could be because the Shannon index takes into account the abundance and evenness of bacteria communities, while the PD incorporates phylogenetic difference between species. Thus, the hepatopancreas had a significant evenness diversity (Shannon) with a lower phylogenetic distance, suggesting a selective pressure of this organ towards the selection of species closely-related to each other. Next, we observed a substantial diversity (Shannon and PD) in the intestine with stool residues compared to the clean intestine. An explanation for this behavior is that the remaining feces in the intestine have a significant diversity that is lost when the intestine is empty (clean intestine), thus, skewing the intestinal diversity when removing them. Furthermore, the effect of the organ in shaping the overall microbiota was stronger when we compared only the adult (Fig. S11a), or juvenile (Fig. S11b) samples separately, suggesting that each developmental stage has a microbiota specific for each organ. This result is in agreement with other studies that also suggest that the gut microbiota was significantly distinct over shrimp developmental stages (Xiong et al., 2017b).

There has been an increased interest in the effect that different diet composition has on the shrimp microbiota and how this could improve the metabolism, growth and health status of this organism. Concerning the feeding intake, we only compared 40 samples in which the diet was specified in the original study, and we found that diversity was higher in shrimps from wt, contrary to the reported for the starlet fish using DGGE (Bacanu & Oprea, 2013). In our meta-analysis, we analyzed the sequencing data of two studies that focused on the effect of carbohydrates (Qiao et al., 2017) and lipids (Zhang et al., 2014b) on the shrimp microbiota. With the lipid supplements, the authors reported a high abundance in Proteobacteria and Tenericutes. These phyla were also two of the most abundant in our analysis; however, they were not related exclusively to the samples with lipids diet. They also do not report significant differences in the microbiota composition between the lipid sources; in contrast, we found a differential enrichment of Shewanella in the samples fed with beef tallow and of Agrobacterium in the samples fed with SBF. Shewanella was a genus significantly enriched in beef-tallow diet, and this is to be expected because this bacteria is one of the most efficient for the metabolism of fatty-acids (Nichols & McMeekin, 2002; Interaminense et al., 2018), coincidently, the metabolism of fatty acids is an enriched function in the stool samples (Fig. S9d). On the other hand, the study that analyzes the effect of carbohydrates also reported a high abundance of Proteobacteria in all their groups and Rhodobacter in one group. Coincidently, our analysis of abundances also showed a significant enrichment of Proteobacteria and Rhodobacter and other genera such as Lactococcus, Paracoccus, and Pseudomonas which were not reported in the original study (Qiao et al., 2017). Interestingly, Lactococcus was reported as an effective probiotic in fishes boosting the immune system and making more efficient the food intake (Dawood et al., 2016).

The lifestyle conditions were the strongest biological factor impacting the microbiota structure of marine samples. Our results showed that wild-type samples were the most diverse and that Vibrionaceae order drove the clustering of those samples, while Enterobacteriaceae and Rhodobactereaceae were the most critical taxa for clustering the farm and laboratory samples. Interestingly, a study in *P. monodon*, also reports higher diversity for wild-type as compared to cultured shrimps (Rungrassamee et al., 2014). These results are in agreement with the previous observations of Cornejo-Granados et al. (2017) in which they also found that wild-type shrimp microbiota is very different than the microbiota of shrimps under cultured conditions. In our meta-analysis, it is not clear the contribution that genetics have in marine shrimps to shape the microbiota composition, although, this can be due to the variation introduced by technical and analysis protocols or by differences in sample size. Although, it is important to note that samples from several shrimp species are too small to draw reliable conclusions about that.

Finally, the developmental stage was also an important factor that shapes the shrimp microbiota (Table 2). The developmental stage with the highest diversity was the adult, followed by juvenile, larvae, and post-larvae, contrary to some studies that report a higher diversity for post-larvae than juvenile (Rungrassamee et al., 2013). The taxonomy of all these groups revealed that Proteobacteria, Bacteroidetes, and Firmicutes were the most abundant phyla for all shrimp developmental stages (Fig. S2a), which is consistent with previous reports in *L. vannamei* and *P. monodon* (Zhang et al., 2014a; Huang et al., 2016; Zheng et al., 2016; Rungrassamee et al., 2013). Particularly in larvae samples, the most abundant family was Enterobacteriaceae, possibly due to the use of whole larvae in the original study (Pangastuti et al., 2010). The authors found that the fecal matter in the larval intestine could be contributing to the high abundance of Enterobacteriaceae, which is in agreement with the observed in our meta-analysis. At this early developmental stage, it is possible that the observed bacterial communities originated mainly from the water, since the shrimp larvae is a filter feeder (Pangastuti et al., 2010). Also, as the immune system reaches full development and the surface of the digestive tract increases, it is possible that the resident bacterial communities become limited with the increase of shrimp developmental stage. Moreover, the enrichment of Aeromonadaceae, Mollicutes and Rhodobactereaceae we observed in juvenile shrimps, and of Vibrionaceae and Pseudomonadaceae in adults is consistent with previous studies in *L. vannamei* (Huang et al., 2016; Moss, LeaMaster & Sweeney, 2000).

We found an enrichment of Caldilinea in all marine shrimps under farm conditions, which is in agreement with the high abundance previously observed of this genus for *L. vannamei* under intensive cultured conditions (Gainza et al., 2017). The denitrification activity characterizes this bacterium, and interestingly it has been found with high abundance in landfills under chemical-stressed conditions (Wu et al., 2017). Given the constant manipulation of farm conditions, the enrichment of this bacterium could be associated with the presence and constant degradation of nitrogen-compounds. Furthermore, we observed an enrichment of Fusobacterium in wt samples, contrary to the reported in *P. monodon*, where this bacteria was found only in domesticated samples (Rungrassamee et al., 2014). In adult shrimps, the significantly enriched genera were Vibrio, Photobacterium, and Fusibacter, which have been previously reported as enriched in adult shrimps of *P. monodon* and *L. vannamei* (Shakibazadeh et al., 2012; Rungrassamee et al., 2014; Cornejo-Granados et al., 2017). On the other hand, Shewanella was found enriched in juvenile shrimps, which is in agreement with the high abundance reported for this genus in juvenile *P. monodon* (Shakibazadeh et al., 2012). We found an enrichment of Candidatus Aquiluna and Microbacterium in larvae shrimps, and both genera are members of the Microbacteriaceae family, which has been previously observed with a high abundance at larvae of *L. vannamei* (Zheng et al., 2017). Regarding the organ, the enriched presence of Vibrio in the intestine and Pseudomonas and Acinetobacter in hepatopancreas also has been previously observed in *L. vannamei* (Cornejo-Granados et al., 2017). Furthermore, the enrichment of the abundance of Shewanella in the intestines has been previously reported for *P. monodon* (Shakibazadeh et al., 2012). Additionally, another genus that we found enriched in the intestine samples were Lactococcus, which is considered as an effective probiotic in fish boosting the immune system and making more efficient the food intake (Dawood et al., 2016). All the genera that we found differentially enriched according to biological factors could be considered as biomarkers for lifestyle, organ, developmental stage, and diet for healthy marine shrimps.

The shrimp health status seems to have the lowest impact on the microbiota structure. For the health status, the most significant diversity was for healthy shrimps, which is in agreement with previous reports (Ringøet al., 2015; Cornejo-Granados et al., 2017). The loss of diversity in diseased samples has been previously reported in the stomach (Chen et al., 2017b), hepatopancreas (Cornejo-Granados et al., 2017), intestine (Cornejo-Granados et al., 2017; Rungrassamee et al., 2016; Xiong, Zhu & Zhang, 2014) and whole larvae (Zheng et al., 2017), independently of the type of disease such as EMS/AHPND (Chen et al., 2017b; Cornejo-Granados et al., 2017), *Vibrio harveyi* infection (Rungrassamee et al., 2016), and others (Zheng et al., 2017; Xiong, Zhu & Zhang, 2014). The early mortality syndrome (EMS), also known as acute hepatopancreatic necrosis disease (AHPND) is a condition associated with the presence of toxins Pir A/B carried by some strains of *V. parahaemolyticus* and that typically affects the hepatopancreas of shrimp postlarvae frequently causing 100% mortality (De Schryver, Defoirdt & Sorgeloos, 2014; Lee et al., 2015). The opportunistic marine pathogen *Vibrio parahaemolyticus* becomes virulent by acquiring a plasmid that expresses the toxin (Lee et al., 2015).

The functional capacity of shrimp microbiota was predicted by PICRUSt. The results suggest that the microbial communities present in each organ perform functions that are significantly different from one organ to another. Interestingly, the clean intestine showed fewer enriched functions than the stool and complete intestine samples, indicating that the bacterial communities present in the feces have a bigger functional contribution than the bacteria attached to the intestine mucosa. In contrast, the developmental stage and lifestyle factors show less differential microbial functions, suggesting that these two biological factors maintain similar metagenome functions. In this regard, several studies also revealed that shrimp microbial functions varied at different culture stages (Zeng et al., 2017), host health status (Cornejo-Granados et al., 2017; Hou et al., 2018), cultural enclosure ecosystems (Hou et al., 2017), host phylogeny (Tzeng et al., 2015), and among cultered and wild-type shrimps (Cornejo-Granados et al., 2017). The approach used in this meta-analysis was a reference mapping protocol, implicating that we only consider the reads that had a 97% sequence similarity with a 16S rRNA genes of the GreenGenes database, limiting the analysis to only known bacteria reported in GreenGenes. We found that approximately 37.9% of the total reads were identified at phylum, class, order, family genus or species level in this database, implicating that 62.1% of reads were unknown, which is consistent with a previous study of shrimp microbiota (Cornejo-Granados et al., 2017). Indeed a reference mapping against SILVA database at 97% sequence similarity also showed that 60.5% of the total reads were unknown. Thus, a more in-depth characterization of the shrimp microbiota is necessary using de novo clustering methods (not reference based) to identify the novel diversity that is unique to the shrimp microbiota. All the functional prediction showed that shrimp microbiota functions significantly varied at different lifestyles, developmental stages, organs, and diets.

## Conclusions

This study aimed to systematically analyze how the shrimp microbiota diversity and function is influenced by different technical and biological factors using a consistent set of bioinformatics methods to avoid this technical bias. After analyzing 199 samples from 16 studies, we observed that despite the high impact that technical and analysis protocols had on the microbiota structure, host factors such as lifestyle, organ, and developmental stage are sufficiently robust to significantly group the samples. The ANOSIM revealed that the environment (marine or freshwater) is the most important biological factor that modulates the shrimp microbiota, showing that freshwater samples have more bacterial diversity than shrimps from a marine environment. Aside from technical factors, the ANOSIM and PERMANOVA analyses agree that all samples of marine shrimps were also significantly grouped by lifestyle, followed by organ and developmental stage (Fig. 6), demonstrating that biological factors significantly shape the structure and function of the shrimp microbiota. However, further studies are needed for a better understanding of the role that these biological factors have on the shrimp microbiota including a more significant number of samples and also including other shrimp species using the same technical and analysis protocols. Our results reinforce the general idea that primers and targeted 16S rRNA hypervariable regions have a substantial impact on the characterization of the shrimp microbiota. In this meta-analysis, we integrated a large number of sequenced samples from different shrimp studies helping us to determine the factors that drive and shape the microbiota structure and function in a non-model organism.

In the search for sequencing data from shrimp microbiota, we found several limitations: (i) only the 60% of the 16S rRNA sequencing data is publically available, and (ii) there were several sequencing data deposited in a single file that includes samples from different conditions making it impossible to obtain the data for each sample. Thus, we strongly recommend researchers to deposit the sequencing data of any shrimp microbiota study to public databases and correctly separate them by sample type, allowing others to obtain more powerful conclusions with large sample size, diverse species, sampling regions, etc. for the benefit of the research on the shrimp microbiota and microbiome fields.

##  Supplemental Information

10.7717/peerj.5382/supp-1 Supplemental Information 1 PRISMA Checklist

10.7717/peerj.5382/supp-2 Figure S1 PRISMA flowchart diagram depicting the search protocol and workflow for systematic review and meta-analysis PRISMA: Preferred Reporting Items for Systematic reviews and Meta-Analyses.

10.7717/peerj.5382/supp-3 Figure S2 Taxonomy diversity and relative abundance for all analyzed samples (A) phylum level, (B) family level. All samples were grouped by species and shrimp origin.

10.7717/peerj.5382/supp-4 Figure S3 Alpha diversity rarefaction curves All curves were calculated at the maximum depth of 1,108 reads per sample. (A) Phylogenetic Diversity (PD), (B) Shannon Index, and (C) Observed OTUs.

10.7717/peerj.5382/supp-5 Figure S4 Biplot PCoA analysis of the microbiota composition of marine and freshwater samples The gray spheres superimposed on the PCoA plot indicate the most abundant bacterial families that drive clustering between marine and freshwater samples. The size of the spheres represents the mean relative abundance of the respective taxon.

10.7717/peerj.5382/supp-6 Figure S5 Alpha diversity index Boxplots indicating the Shannon index for all samples grouped by lifestyle, host, diet, and health status categories. A sequence depth of 1,108 reads and 10,000 iterations were used to calculate the Shannon index value.

10.7717/peerj.5382/supp-7 Figure S6 Biplot PCoA analysis of the microbiota composition of marine samples The gray spheres superimposed on the PCoA plot indicate the most abundant bacterial families that drive clustering between (A) samples tagged by lifestyle and (B) samples tagged by developmental stage. The size of the spheres represents the mean relative abundance of the respective taxon.

10.7717/peerj.5382/supp-8 Figure S7 Beta diversity analysis of marine samples Unweighted principal coordinate analysis (PCoA) of UniFrac distances with samples tagged by health-status.

10.7717/peerj.5382/supp-9 Figure S8 Beta diversity analysis of *L. vannamei* samples Unweighted principal coordinate analysis (PCoA) of UniFrac distances with samples tagged by (A) lifestyle, (B) organ and (C) developmental stage.

10.7717/peerj.5382/supp-10 Figure S9 LEfSE results of enriched predicted functions for all marine samples We used PICRUSt to predict the functions based on the 16S rRNA sequencing data, then we performed a LEfSe analysis to reveal the most enriched functions in the following categories: (A) lifestyle, (B) organ, (C) developmental stage and (D) diet. The graph shows the log10 LDA score for each classification.

10.7717/peerj.5382/supp-11 Figure S10 Beta diversity analysis of marine and freshwater samples Unweighted principal coordinate analysis (PCoA) of UniFrac distances with all 199 samples tagged by (A) paper, (B) primers and (C) hypervariable region.

10.7717/peerj.5382/supp-12 Figure S11 Beta diversity analysis marine samples Unweighted principal coordinate analysis (PCoA) of UniFrac distances with (A) adult marine samples and (B) juvenile marine samples tagged by organ.

10.7717/peerj.5382/supp-13 Table S1 Terms used for the systematic paper search in Scopus

10.7717/peerj.5382/supp-14 Table S2 Original accession number of the 199 samples used for the meta-analysis, number of reads after quality filtering and number of reads taxonomically assigned with QIIME In red we show the samples eliminated from the analysis due to the low number of assigned reads.

10.7717/peerj.5382/supp-15 Table S3 Articles used for shrimp microbiota meta-analysis (30 articles) In red are shown the studies that mixed sequences from all samples in one file.

10.7717/peerj.5382/supp-16 Table S4 Sample type with available sequenced data for marine and freshwater samples All data were collected from the original study for each sample. ****

10.7717/peerj.5382/supp-17 Table S5 Technical and biological factors associated with the microbial structure of shrimp microbiota The impact was measured using Anosim (*R* value) and PERMANOVA with the Adonis function (*F* and *R*^2^ values) of Bray–Curtis distances. For each analysis, we performed 1,000 permutations to obtain the *p*-value.

10.7717/peerj.5382/supp-18 Supplemental Information 2 Additional response The rationale for conducting this meta-analysis.

## Additional Information and Declarations

### Data Availability

The following information was supplied regarding data availability: All the sequencing data used in this meta-analysis are described in Table S2.
