# Supplementary figures and images for: A meta-analysis reveals the environmental and host factors shaping the structure and function of the shrimp microbiota
Source: PeerJ. 2018 Aug 10;6:e5382. doi: 10.7717/peerj.5382 (PMC6089209; doi:10.7717/peerj.5382)

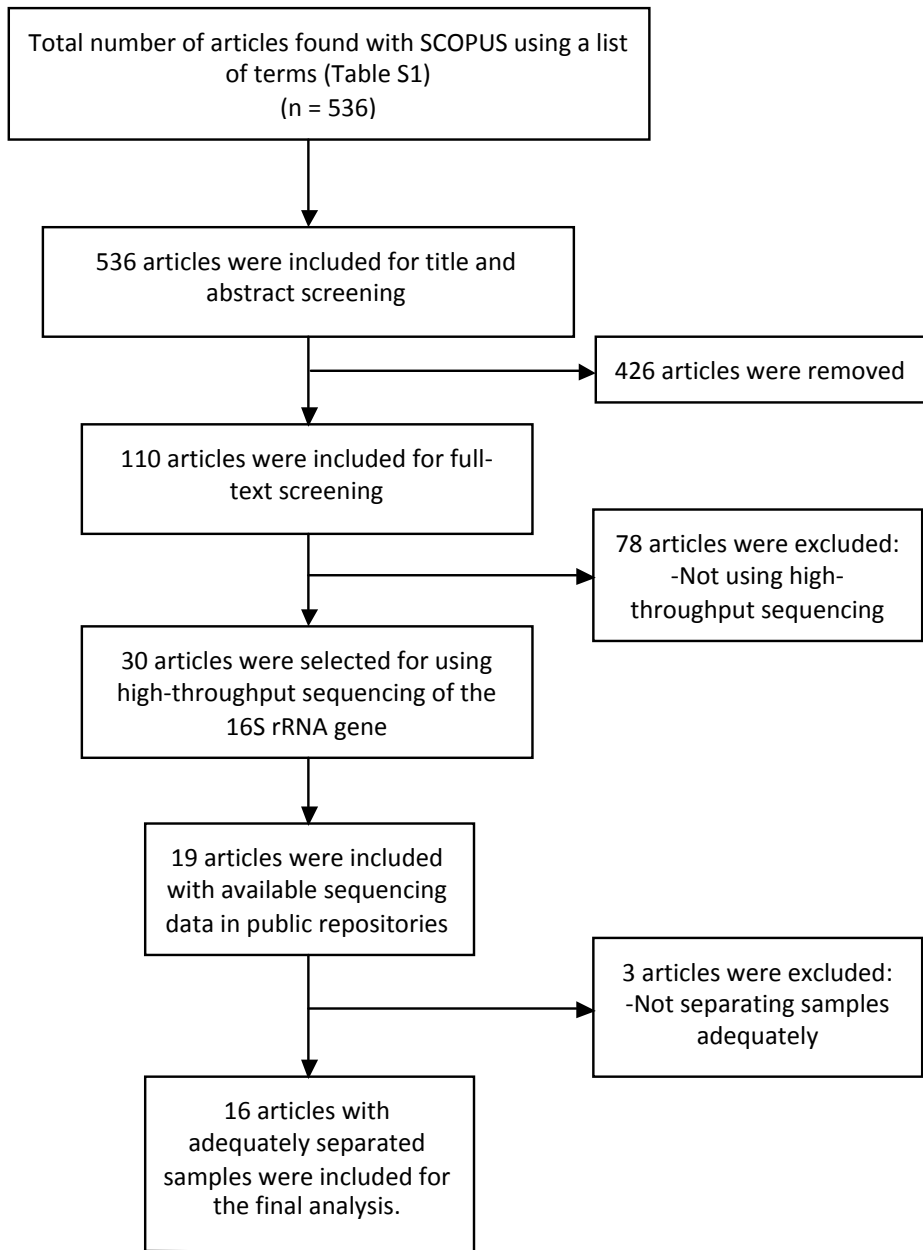

Supplement: Figure S1 — PRISMA: Preferred Reporting Items for Systematic reviews and Meta-Analyses. [file peerj-06-5382-s002.pdf]

a

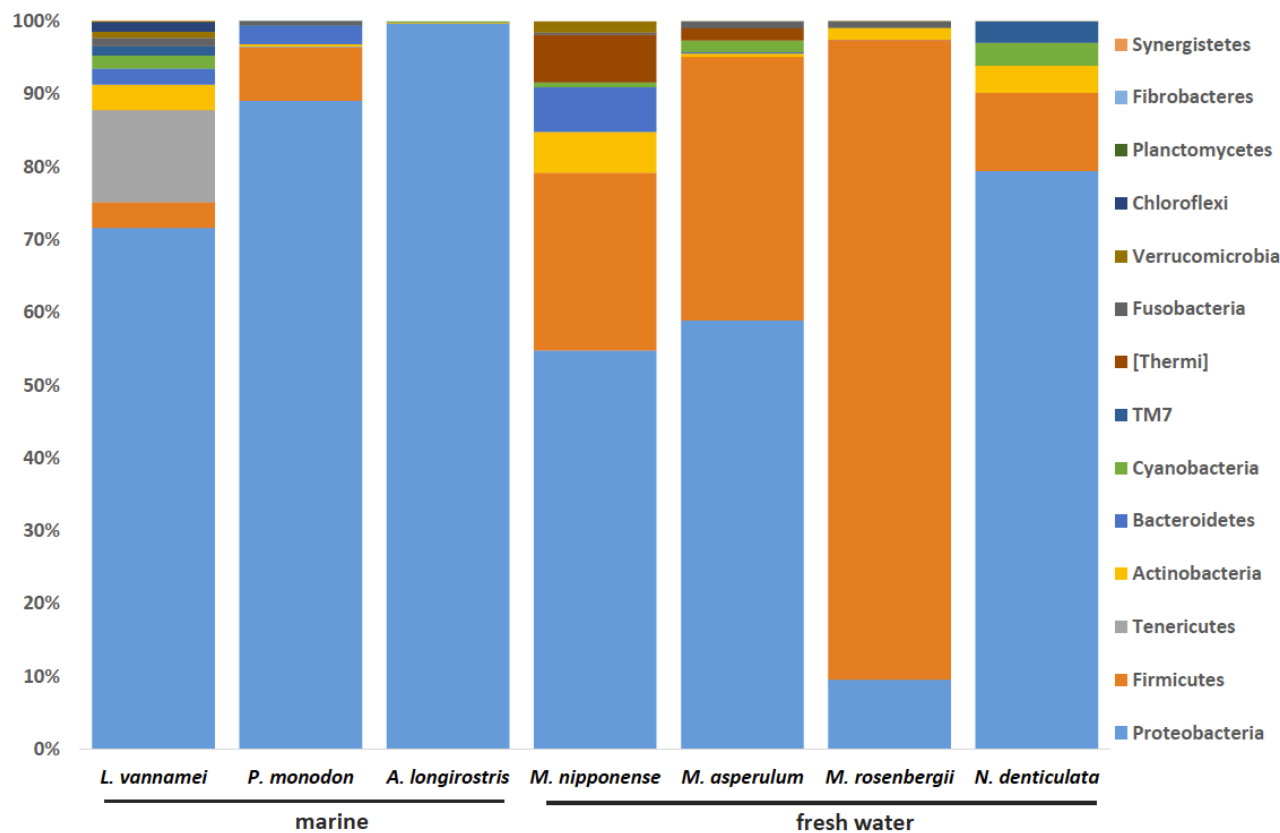

b

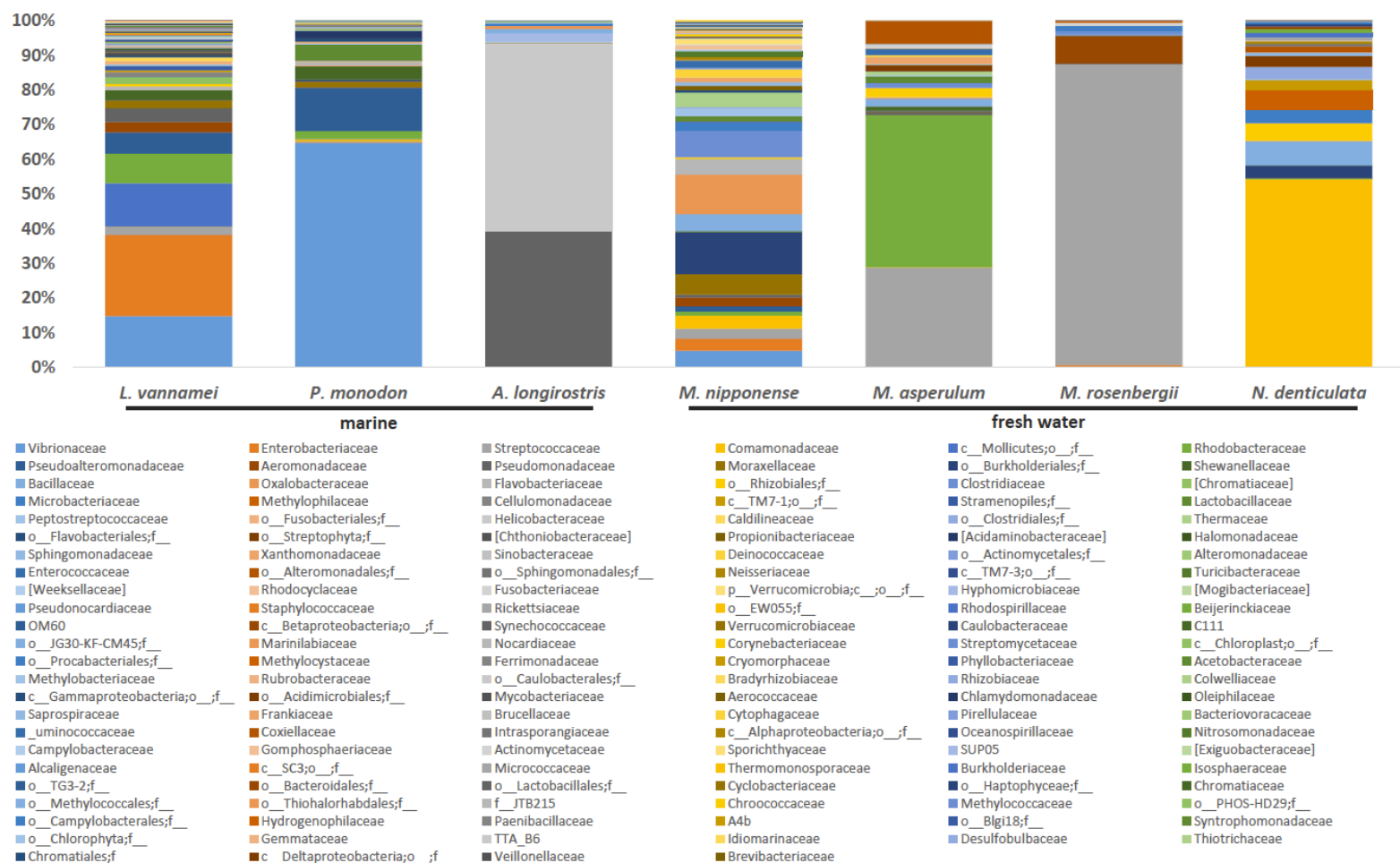

Supplement: Figure S2 — (A) phylum level, (B) family level. All samples were grouped by species and shrimp origin. [file peerj-06-5382-s003.pdf]

**a**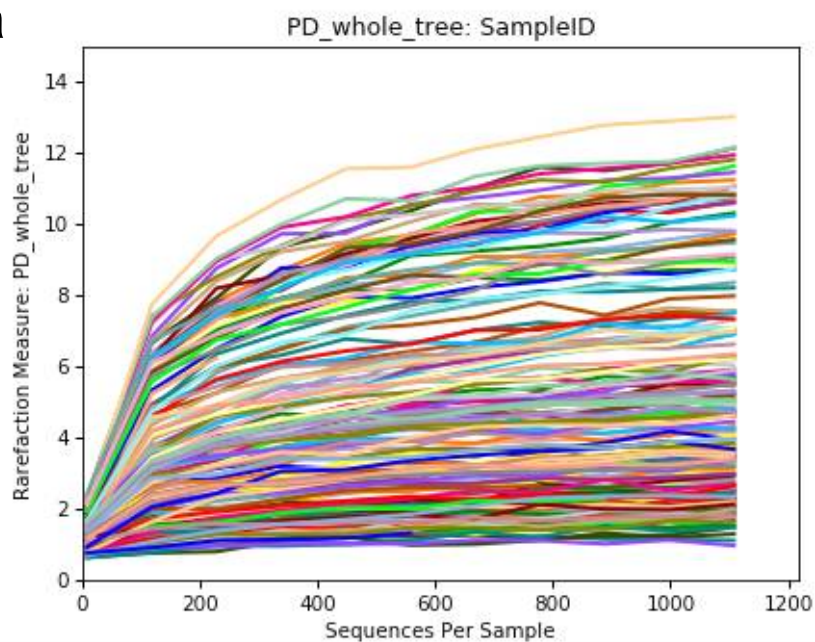**b**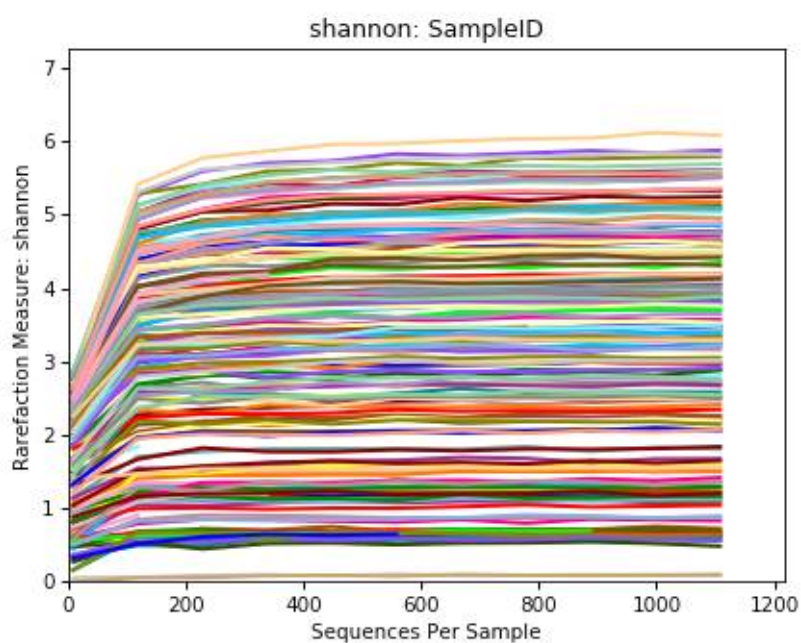**c**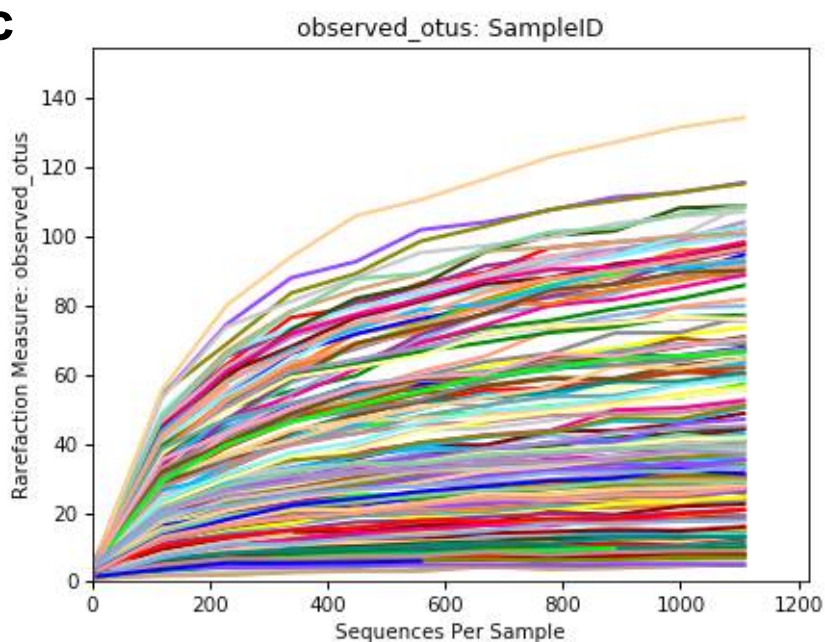

Supplement: Figure S3 — All curves were calculated at the maximum depth of 1,108 reads per sample. (A) Phylogenetic Diversity (PD), (B) Shannon Index, and (C) Observed OTUs. [file peerj-06-5382-s004.pdf]

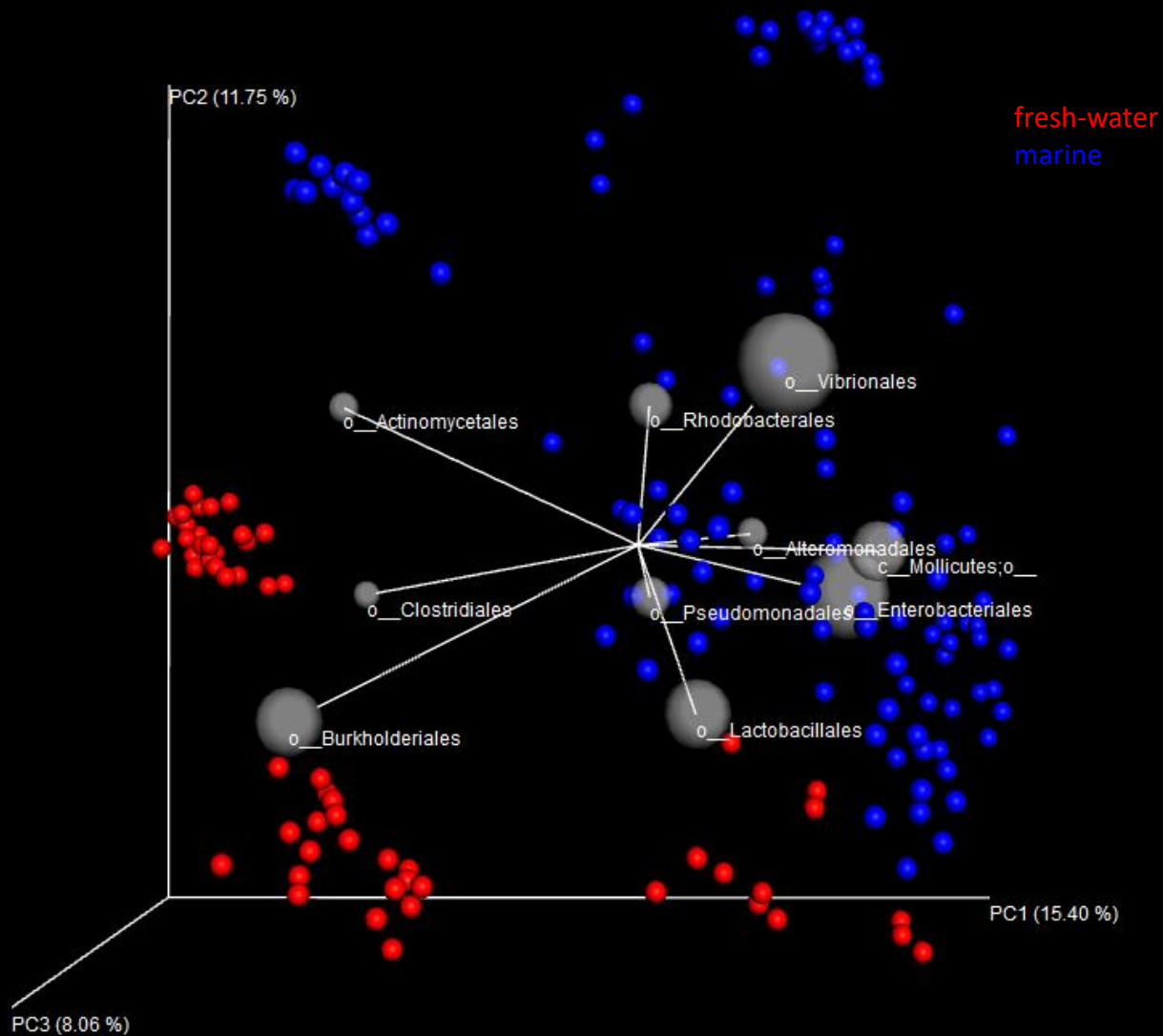

Supplement: Figure S4 — The gray spheres superimposed on the PCoA plot indicate the most abundant bacterial families that drive clustering between marine and freshwater samples. The size of the spheres represents the mean relative abundance of the respective taxon. [file peerj-06-5382-s005.pdf]

**a**

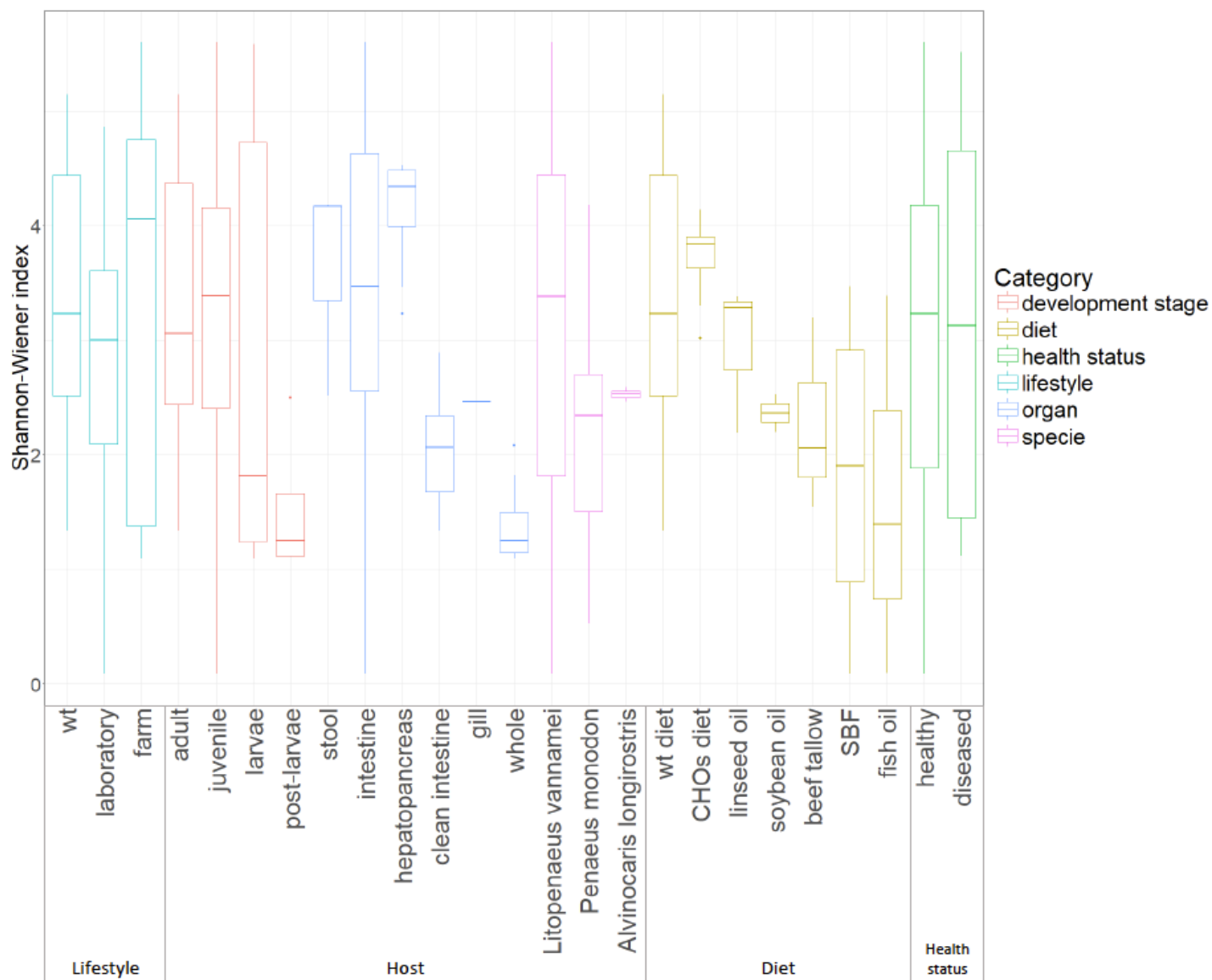

**b**

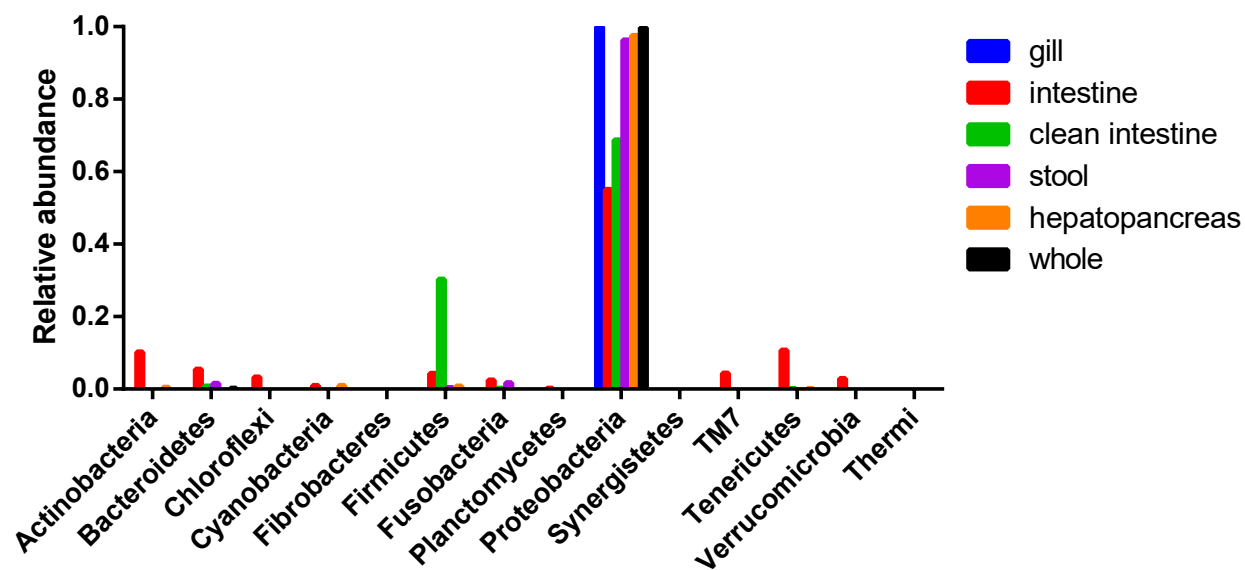

Supplement: Figure S5 — Boxplots indicating the Shannon index for all samples grouped by lifestyle, host, diet, and health status categories. A sequence depth of 1,108 reads and 10,000 iterations were used to calculate the Shannon index value. [file peerj-06-5382-s006.pdf]

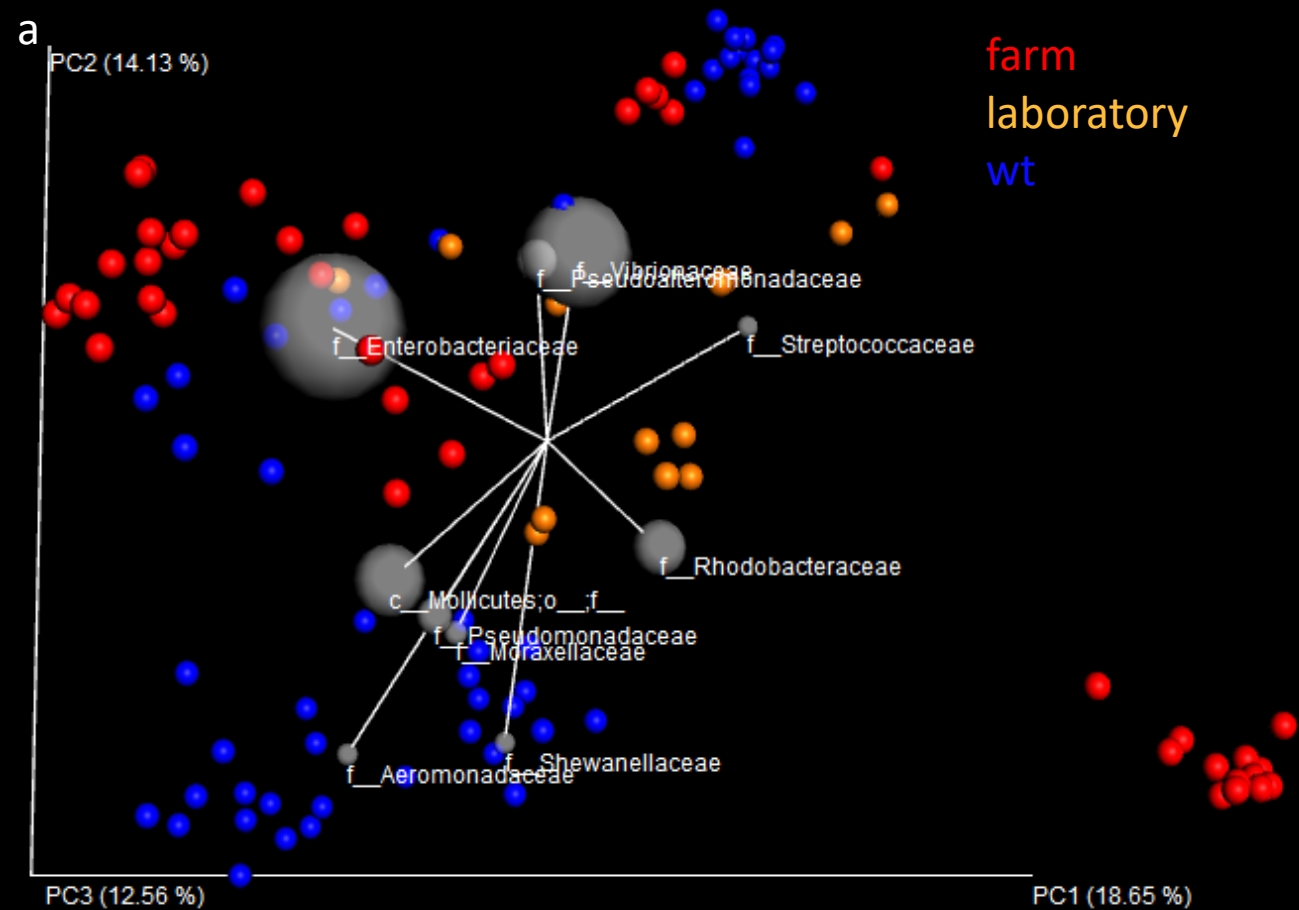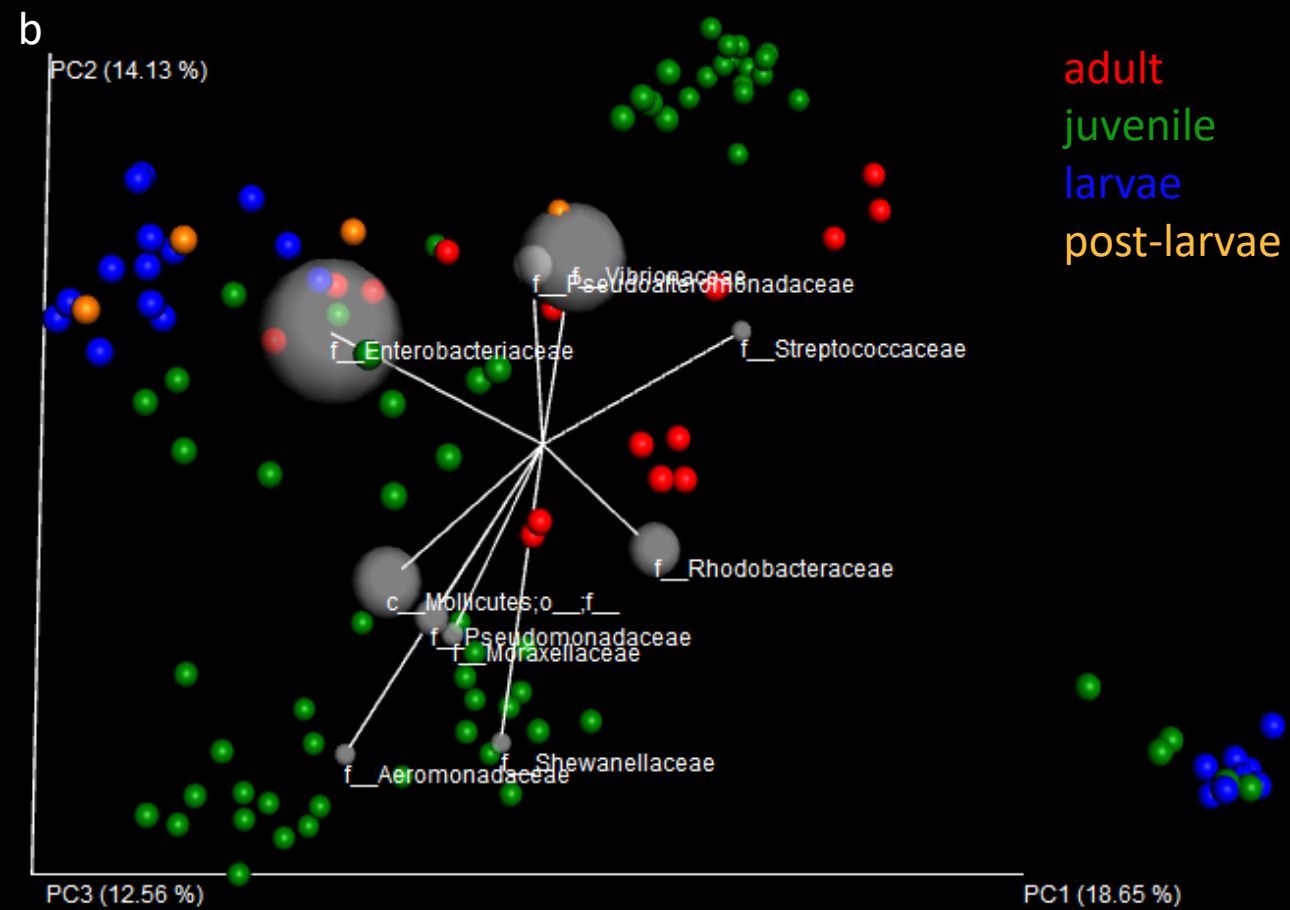

Supplement: Figure S6 — The gray spheres superimposed on the PCoA plot indicate the most abundant bacterial families that drive clustering between (A) samples tagged by lifestyle and (B) samples tagged by developmental stage. The size of the spheres represents the mean relative abundance of the respective taxon. [file peerj-06-5382-s007.pdf]

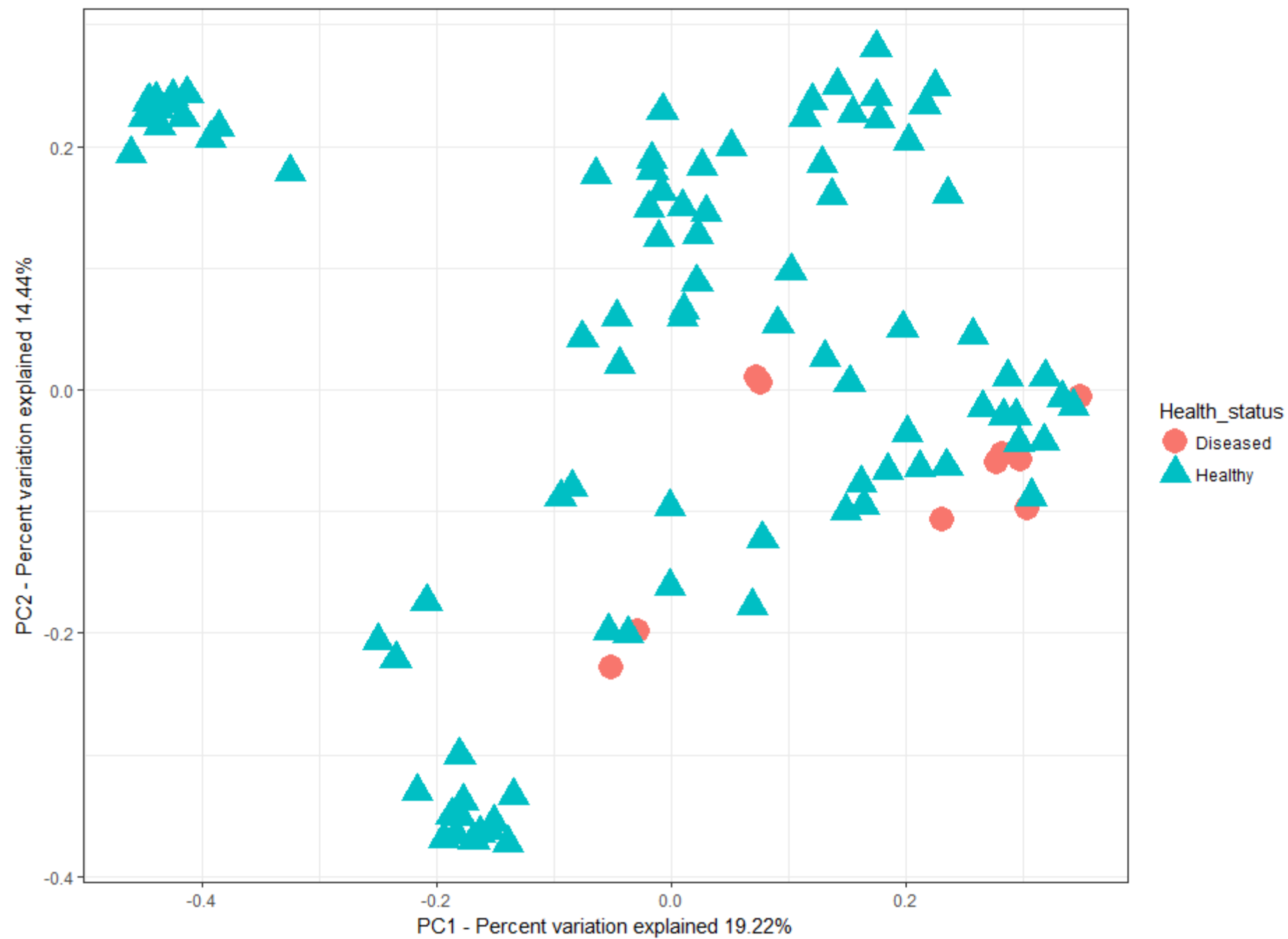

Supplement: Figure S7 — Unweighted principal coordinate analysis (PCoA) of UniFrac distances with samples tagged by health-status. [file peerj-06-5382-s008.pdf]

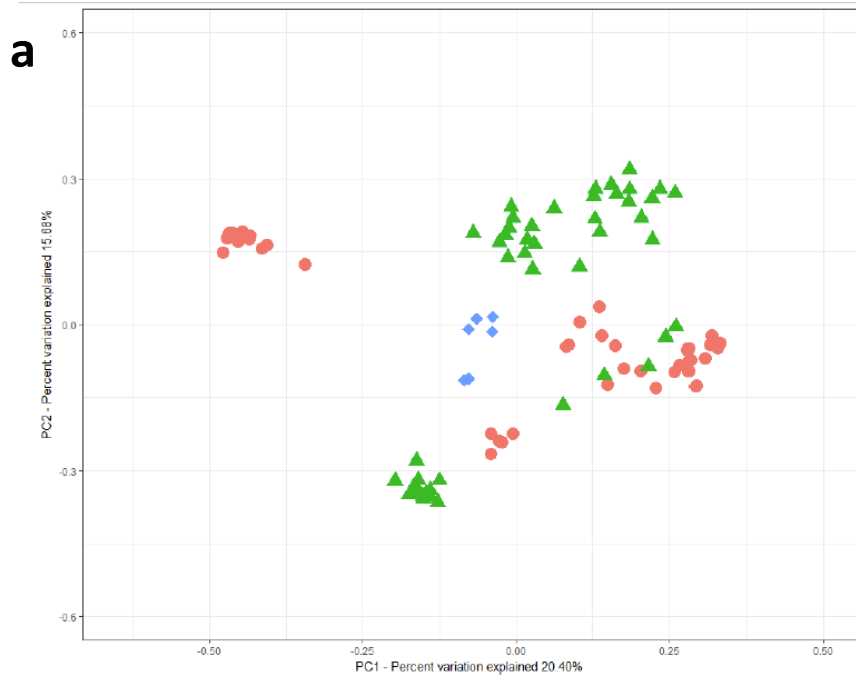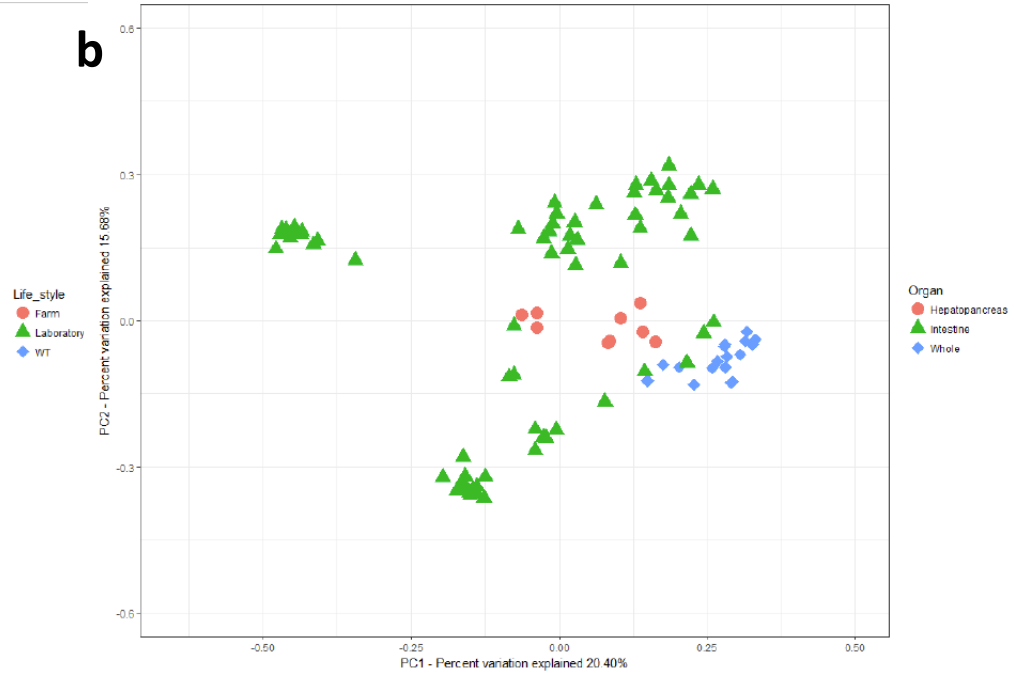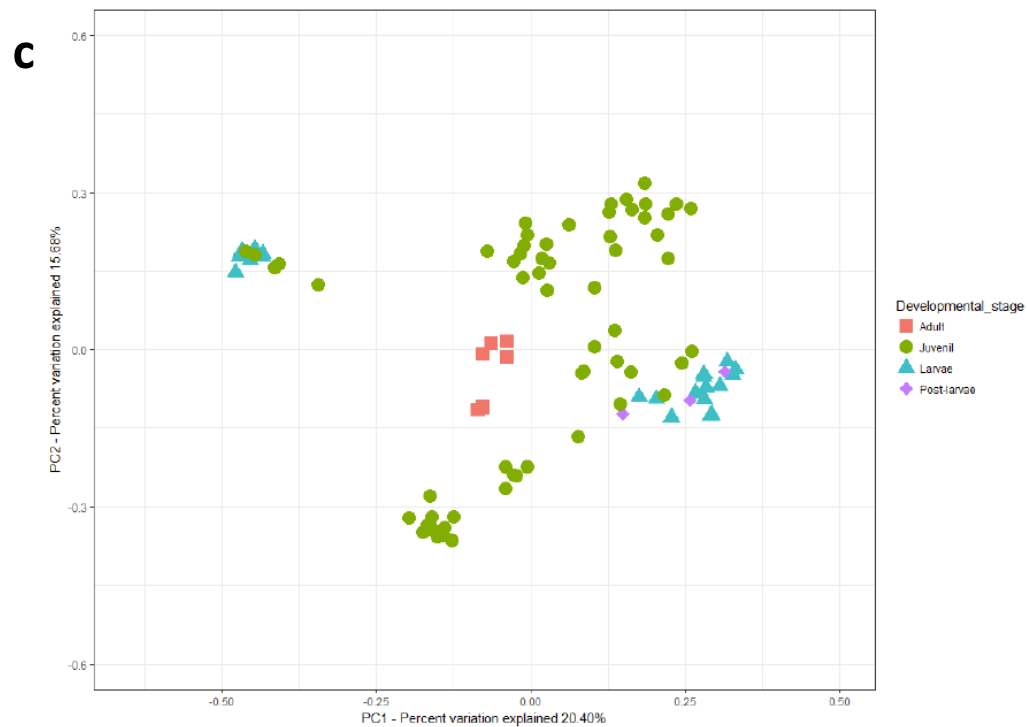

Supplement: Figure S8 — Unweighted principal coordinate analysis (PCoA) of UniFrac distances with samples tagged by (A) lifestyle, (B) organ and (C) developmental stage. [file peerj-06-5382-s009.pdf]

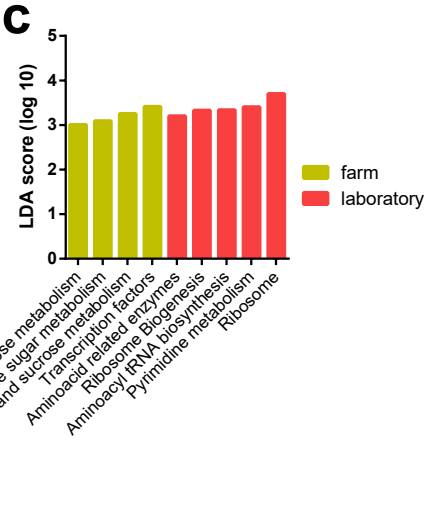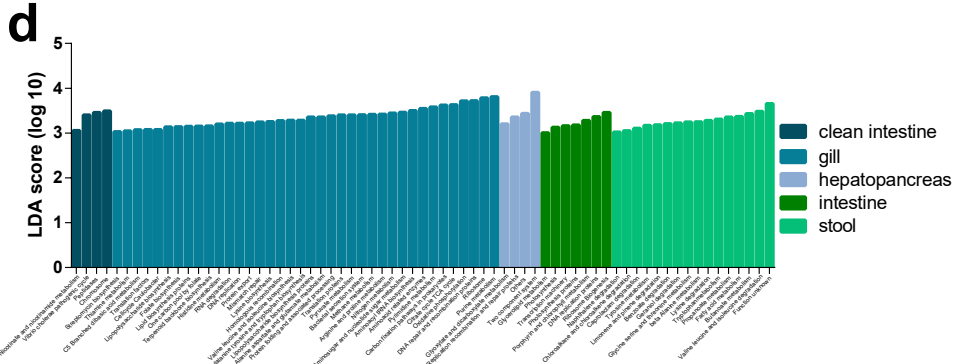

Supplement: Figure S9 — We used PICRUSt to predict the functions based on the 16S rRNA sequencing data, then we performed a LEfSe analysis to reveal the most enriched functions in the following categories: (A) lifestyle, (B) organ, (C) developmental stage and (D) diet. The graph shows the log10 LDA score for each classification. [file peerj-06-5382-s010.pdf]

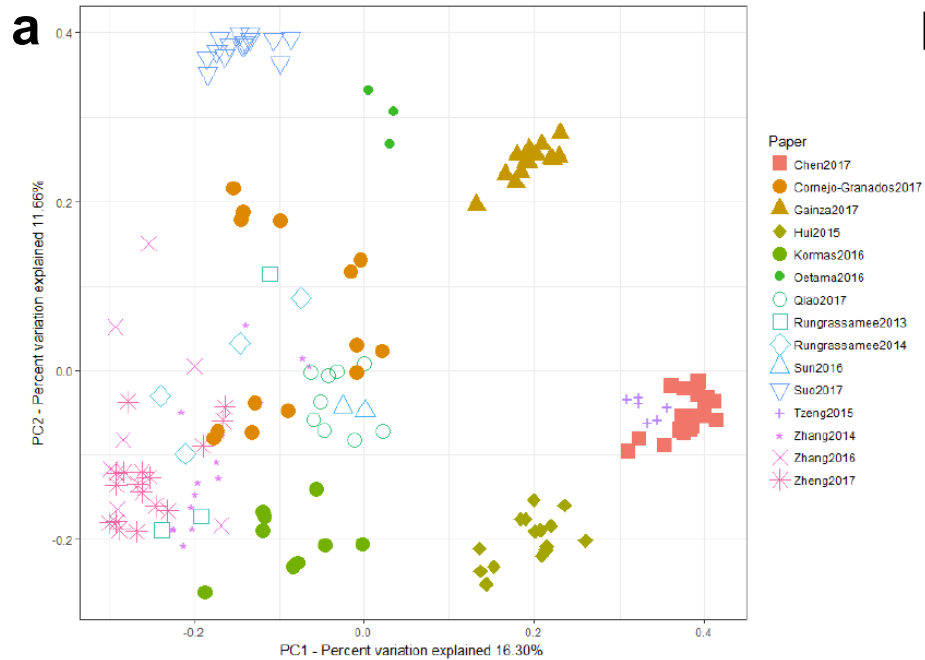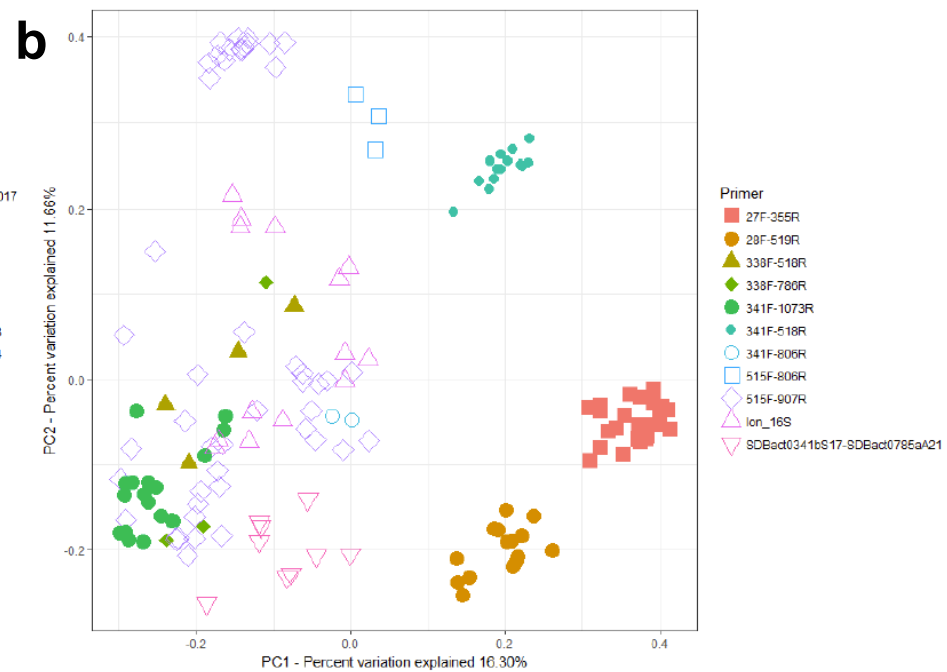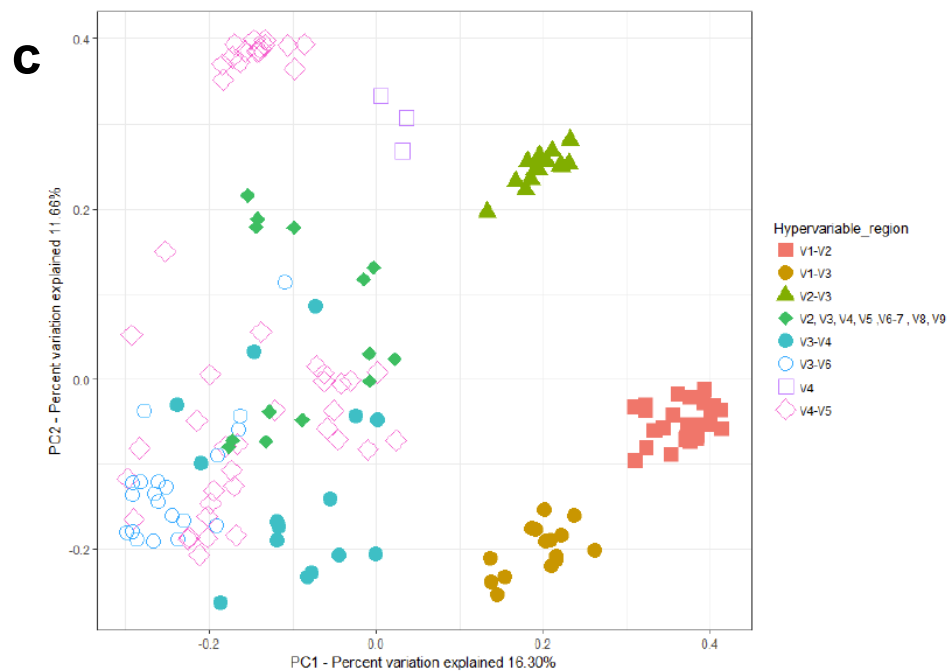

Supplement: Figure S10 — Unweighted principal coordinate analysis (PCoA) of UniFrac distances with all 199 samples tagged by (A) paper, (B) primers and (C) hypervariable region. [file peerj-06-5382-s011.pdf]

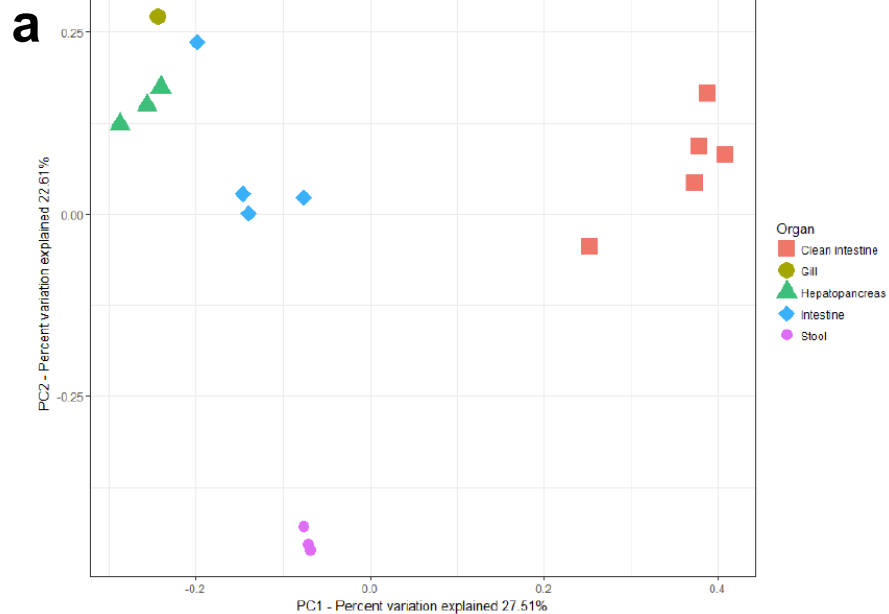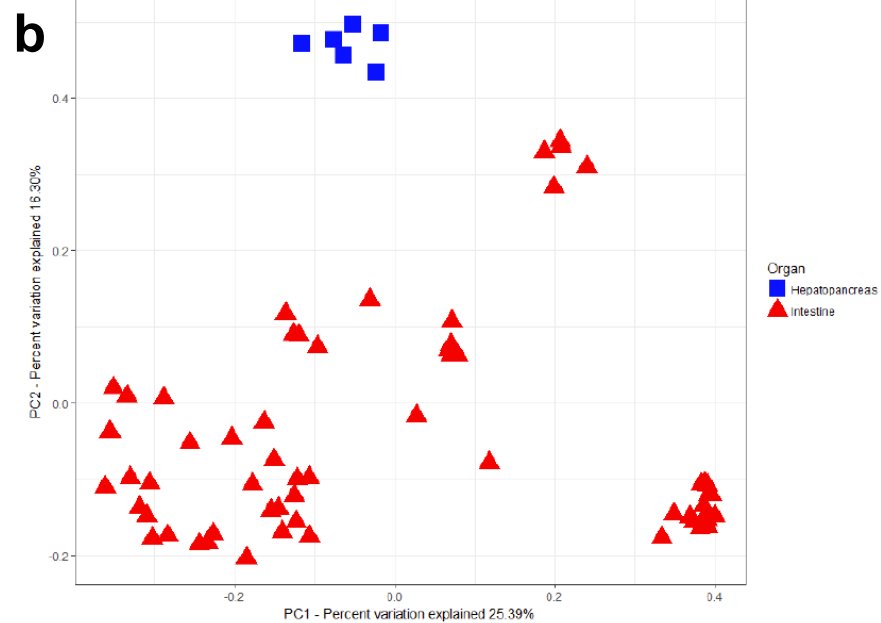

Supplement: Figure S11 — Unweighted principal coordinate analysis (PCoA) of UniFrac distances with (A) adult marine samples and (B) juvenile marine samples tagged by organ. [file peerj-06-5382-s012.pdf]
