# Supplementary material for: A meta-analysis reveals the environmental and host factors shaping the structure and function of the shrimp microbiota
Source: PeerJ. 2018 Aug 10;6:e5382. doi: 10.7717/peerj.5382 (PMC6089209; doi:10.7717/peerj.5382)
Supplement: Table S1 [file peerj-06-5382-s013.pdf]

Table S1: Terms used for the systematic paper search in Scopus.

shrimp microbiome
shrimp microbiota
vannamei microbiome
vannamei microbiota
prawn microbiome
prawn microbiota
bacterial community and shrimp
bacterial community and prawn
Bacterial biota and shrimp
Bacterial biota and prawn
bacterial communities and prawn
bacterial communities and shrimp
microbial communities and shrimp
microbial communities and prawn
microflora prawn
microflora shrimp
bacterial biota and shrimp
bacterial biota and prawn
bacterial diversity and prawn

bacterial diversity and shrimp
bacterial population and shrimp
bacterial population and prawn
microbiotic shrimp
microbiotic prawn
metagenome shrimp
metagenomic shrimp
metagenome prawn
metagenomic prawn
next generation sequencing and shrimp
next generation sequencing and prawn
bacterial composition and shrimp
bacterial composition and prawn
microbial composition and shrimp
microbial composition and prawn
bacterial composition and shrimp
bacterial composition and prawn
