# Supplementary material for: A meta-analysis reveals the environmental and host factors shaping the structure and function of the shrimp microbiota
Source: PeerJ. 2018 Aug 10;6:e5382. doi: 10.7717/peerj.5382 (PMC6089209; doi:10.7717/peerj.5382)
Supplement: Table S2 — In red we show the samples eliminated from the analysis due to the low number of assigned reads. [file peerj-06-5382-s014.pdf]

Table S2. Original accession number of the 199 samples used for the meta-analysis, number of reads after quality filtering and number of reads taxonomically assigned with QIIME. In red we show the samples eliminated from the analysis due to the low number of assigned reads.

SampleID	Reads with quality ≥Q20	Reads asigned with QIIME
SRR2072206	4,398,093	3,796,396
SRR2072205	2,113,069	1,786,623
SRR2072209	1,575,835	1,416,484
SRR2720402	266,417	207,409
SRR4999939	242,815	166,563
SRR2720405	213,441	133,550
SRR5062153	434,788	118,371
SRR5062186	293,296	78,196
SRR4999941	128,933	75,189
SRR5062169	272,259	73,820
SRR5062154	226,381	73,365
SRR5062174	265,451	72,023
SRR5062177	409,612	69,932
SRR4999938	142,180	69,892
SRR5062167	252,879	69,161
SRR4999940	115,426	64,171
SRR4999945	157,158	63,056
SRR5062181	189,106	61,679
SRR4999934	106,272	59,516
SRR4999935	97,818	56,126
SRR4999944	113,463	52,707
SRR4424562	66,369	51,060
SRR5062178	217,352	49,915
SRR4999946	106,708	47,884
SRR4424555	59,383	45,850
SRR5062175	196,756	45,140
SRR4424551	62,766	44,143
SRR4999943	105,724	42,626
SRR5062180	210,120	42,566
SRR5062182	160,453	41,247
SRR4424563	49,808	41,064
SRR5062161	179,437	40,535

SRR5062189	151,199	40,488
SRR5062165	198,433	39,831
SRR5062164	158,778	39,430
SRR4999936	125,706	39,176
SRR5062185	194,483	38,889
SRR5062183	153,630	34,994
SRR4424552	51,000	33,958
SRR4424557	48,100	33,440
SRR4424558	53,831	31,730
SRR5062188	141,749	31,317
SRR4999942	86,598	31,080
SRR1425398	30,048	28,476
SRR4424550	45,108	28,260
SRR4424561	39,621	26,868
SRR4424564	43,552	26,314
SRR4999947	104,959	25,963
SRR4424554	35,804	25,577
SRR4424559	43,206	23,757
SRR1732361	28,930	22,701
SRR4424556	46,847	20,460
SRR1425397	21,129	18,950
SRR4039040	22,909	18,366
SRR4039033	21,921	17,912
SRR4039035	21,163	17,456
SRR1424469	21,553	17,423
SRR4424553	32,592	17,036
SRR4039029	20,946	16,901
SRR4039038	20,944	16,808
SRR1425402	28,803	16,680
SRR4039034	21,424	16,463
SRR4039031	20,307	16,368
SRR4039050	21,048	16,306
SRR4039037	19,840	16,291
MB2A	19,193	16,110
SRR4039054	19,868	15,895
SRR4039030	19,165	15,793
SRR4039039	19,062	15,764
SRR4039032	18,999	15,690
SRR4039053	19,179	15,597
SRR4039052	19,594	15,495
SRR1980942	40,584	15,403
SRR4039051	19,764	15,007
SRR1425340	25,668	14,914
MB8B	16,970	14,711
SRR1732367	22,539	14,546

SRR4039056	18,548	14,366
SRR1425406	26,815	14,255
SRR1980944	37,154	13,677
SRR1980943	35,364	12,658
SRR2128016	18,951	12,142
SRR1425396	15,339	11,582
SRR2128017	21,567	11,548
SRR4999937	84,006	11,227
MB6B	12,857	11,105
SRR2128005	24,521	10,495
SRR1980946	17,609	10,369
Hg.M.1	27,117	10,296
SRR1980945	21,450	10,268
JX939518	13,130	10,200
MB9B	11,367	9,814
SRR1425393	20,342	9,712
SRR1980947	18,712	9,516
MB5A	10,776	9,153
SRR1425401	25,138	8,793
MB10B	10,150	8,704
Hg.L.1	36,321	8,621
SRR1425400	28,364	8,460
SRR1425404	19,222	8,354
Hp.M.2	22,728	8,077
SRR1425408	10,282	7,676
SRR1732360	25,016	7,570
KF344403	9,952	7,262
SRR2128038	25,753	7,215
KF355928	11,525	6,769
SRR1425341	19,423	6,717
SRR2128015	9,408	6,397
Hp.M.3	24,783	6,153
SRR1425410	24,813	6,036
SRR2128037	23,469	6,035
SRR1732362	23,302	6,022
Hg.L.3	22,162	5,658
SRR2128036	16,651	5,551
Hg.M.2	35,524	4,955
Hg.L.2	31,428	4,909
Fg.M.2	24,031	4,875
SRR5585685	10,723	4,677
SRR2128006	9,786	4,637
SRR2128039	9,470	4,477
SRR5585684	11,326	4,327
SRR1732363	29,018	4,325

SRR1732364	29,018	4,325
SRR1732365	11,283	4,176
SRR5585681	7,725	4,058
MB11C	5,028	4,049
KF334451	5,023	3,956
SRR1732366	16,679	3,921
SRR5585672	10,030	3,757
SRR5585674	6,489	3,691
Fg.M.1	25,085	3,680
MB12C	4,631	3,618
MB3A	4,812	3,526
SRR4424560	13,951	3,513
SRR5585676	7,554	3,502
SRR1425405	27,896	3,464
SRR5585664	7,182	3,461
Fg.L.3	24,187	3,415
JX926388	7,045	3,319
Hp.L.1	19,658	3,197
SRR5585673	8,356	3,075
SRR5585683	5,147	2,954
SRR5585686	4,703	2,727
JX919343	3,055	2,603
KF325238	2,959	2,549
SRR5585678	4,452	2,531
SRR5585667	5,830	2,523
Fg.L.1	28,224	2,496
Hp.E.3	18,255	2,437
Fg.L.2	18,067	2,406
SRR5585670	3,700	2,343
SRR5585665	3,868	2,239
Hp.L.2	21,809	2,219
SRR1425336	19,540	2,162
SRR5585677	3,957	2,153
Fg.M.3	19,004	2,034
SRR1425411	17,814	2,002
KF328420	3,182	1,995
SRR5585687	15,814	1,966
SRR5585682	5,721	1,904
Hg.M.3	20,150	1,895
MB16C	2,177	1,704
Hp.M.1	19,246	1,611
MC17D	2,111	1,502
MB19D	1,659	1,248
Hp.E.2	8,622	1,212
Hp.E.1	19,184	1,190

MB7B	1,601	1,108
SRR1425339	16,064	994
Hp.L.3	14,200	896
SRR5585669	1,724	895
JX941408	1,890	882
MB4A	1,273	753
MB13C	1,225	602
KF329428	1,008	542
KP946571	1,890	415
KP948363	1,672	364
MB1A	1,039	342
KP951735	2,904	337
MB21D	550	224
KP952978	731	151
MB20D	777	143
KP952247	512	140
Hg.E.2	34,637	115
KP953763	465	115
MB14C	260	83
Hg.E.3	24,445	82
KP944681	474	75
KP953298	320	59
KP948831	302	58
MB18D	1,172	50
KP948529	166	45
Fg.E.3	31,016	35
KP946691	120	30
Hg.E.1	35,435	26
KP953903	140	23
MB15C	105	3
Fg.E.1	35,029	2
Fg.E.2	30,678	2
