# Supplementary material for: A meta-analysis reveals the environmental and host factors shaping the structure and function of the shrimp microbiota
Source: PeerJ. 2018 Aug 10;6:e5382. doi: 10.7717/peerj.5382 (PMC6089209; doi:10.7717/peerj.5382)
Supplement: Table S3 — In red are shown the studies that mixed sequences from all samples in one file. [file peerj-06-5382-s015.pdf]

Table S3. Articles used for shrimp microbiota meta-analysis (30 articles). In red are shown the studies that mixed sequences from all samples in one file.

**Reference**	**Hypervariable region**	**Sequencing technology**	**Shrimp specie**	**Country**	**Accession number**
Zheng *et al*., 2017	V3-V6	Roche 454	Litopenaeus vannamei	China	SRP080243
Chen *et al*., 2017	V1-V2	Illumina MiSeq	Macrobrachium nipponense	Taiwan	SRP094102
Suo et al., 2017	V4-V5	Illumina MiSeq	Litopenaeus vannamei	China	SRP091598
Rungranssamme et al., 2014	V3-V4	Roche 454	Penaeus monodon	Tailand	KF329429–KF334451, KF334452–KF344403, KF344404–KF355928, KF322280–KF325238, KF325239–KF328420, KF328421–KF329428
Cornejo-Granados et al., 2017	V2, V3, V4, V5 ,V6-7 , V8, V9	Ion Torrent	Litopenaeus vannamei	Mexico	SRP107821
Gainza et al., 2017	V2-V3	Ion Torrent	Litopenaeus vannamei	Ecuador	SRP092753
Qiao et al., 2017	V4-V5	Illumina MiSeq	Litopenaeus vannamei	China	SRP061605
Sun et al., 2016	V3-V4	Illumina MiSeq	Alvinocaris longirostris	Japan	SRP064953
Tzeng et al., 2015	V1-V2	Roche 454	Macrobrachium asperulum, Macrobrachium nipponense	Taiwan	SRP057429
Oetama et al., 2016	V4	Illumina MiSeq	Penaeus monodon	Indonesia	SRP059721
Zhang et al., 2014	V4-V5	Illumina MiSeq	Litopenaeus vannamei	China	SRP043399
Zhang et al., 2016	V4-V5	Illumina MiSeq	Litopenaeus vannamei	China	SRP051489
Rungranssamme et al., 2013	V3-V6	Roche 454	Penaeus monodon	Tailand	JX919344-JX926388, JX916289-

					JX919343, JX926389-JX939518, JX939519-JX941408.
Rungranssamme et al., 2016	V3-V4	Roche 454	Penaeus monodon	Thailand	KP944208-KP944681, KP948364-KP948529, KP944682-KP946571, KP946572-KP946691, KP946692-KP948363, KP948530-KP948831, KP948832-KP951735, KP953299-KP953763, KP951736-KP952247, KP952248-KP952978, KP952979-KP953298, KP953764-KP953903
Mente E et al., 2016	V3-V4	Roche 454	Macrobranchium resenbergii	Greece	SRR1502207
Cheung M.K et al., 2015	V1-V3	Ion Torrent	Neocaridina denticulata	China	SRR1735538
Zeng S et al., 2017	V4	Illumina HiSeq	Litopenaeus vannamei	China	SRX2946975
Sha Y et al., 2016	V1-V2	Illumina MiSeq	Litopenaeus vannamei	China	SRX1609125
Xiong, Wang et al., 2015	V4	Illumina MiSeq	Litopenaeus vannamei	China	DRA002398
Cui et al., 2017	V4	Illumina MiSeq	Litopenaeus vannamei, Macrobrachium rosenbergii	China	
Xiong, Zhu et al.,	V3-V4	Illumina	Litopenaeus	China	

2017		MiSeq	vannamei		
Xiong, Dai et al., 2017	V3-V4	Illumina MiSeq	Litopenaeus vannamei	China	
Huang Z et al., 2016	V3-V5	Roche 454	Litopenaeus vannamei	China	
Vargas-Albores F et al., 2017	V3-V4	Illumina MiSeq	Litopenaeus vannamei	Mexico	
Chen W-Y et al., 2017	V3–V4	Illumina MiSeq	Litopenaeus vannamei	Taiwan	
Zhou Y et al., 2017	V1-V3	Illumina MiSeq	Litopenaeus vannamei	China	
Li K et al., 2007	-	-	Litopenaeus vannamei	China	
Wen C et al., 2016	V4	Illumina MiSeq	Litopenaeus vannamei	China	
Cardona E et al., 2016	V4	Illumina MiSeq	Litopenaeus vannamei	French Polynesia	
Cowart DA et al., 2017	V3-V4	Roche 454	Rimicaris exoculata	France	
