# Supplementary material for: A meta-analysis reveals the environmental and host factors shaping the structure and function of the shrimp microbiota
Source: PeerJ. 2018 Aug 10;6:e5382. doi: 10.7717/peerj.5382 (PMC6089209; doi:10.7717/peerj.5382)
Supplement: Table S4 — All data were collected from the original study for each sample. [file peerj-06-5382-s016.pdf]

Table S4. Sample type with available sequenced data for marine and freshwater samples. All data was collected from the original study for each sample.

	**marine samples**	**freshwater samples**
**habitats**	wt, farm, laboratory	wt, farm, laboratory
**organs**	intestine, clean intestine, gill, hepatopancreas, whole	intestine, clean intestine, hepatopancreas, foregut
**growth-stages**	adult, larvae, post-larvae, pre-adult	adult
**diets**	wt, CHOs, soybean oil, beef tallow, linseed oil, fish oil, SBF	wt
**species**	*L. vannamei, P. monodon, A. longirostris*	*M. nipponense, M. rosenbergii, M. asperulum, N. Denticulata*
**health status**	healthy and diseased	healthy
