# Supplementary material for: A meta-analysis reveals the environmental and host factors shaping the structure and function of the shrimp microbiota
Source: PeerJ. 2018 Aug 10;6:e5382. doi: 10.7717/peerj.5382 (PMC6089209; doi:10.7717/peerj.5382)
Supplement: Table S5 — The impact was measured using Anosim (R value) and PERMANOVA with the Adonis function (F and R2 values) of Bray–Curtis distances. For each analysis, we performed 1,000 permutations to obtain the p-value. [file peerj-06-5382-s017.pdf]

**Table S5. Technical and biological factors associated with the microbial structure of shrimp microbiota.** The impact was mesured using Anosim (R value) and PERMANOVA with the adonis function (F and $R^2$ values) of Bray-Curtis distances. For each analysis we performed 1,000 permutations to obtain the p value.

| Bray-Curtis | | | | | | | |
|---|---|---|---|---|---|---|---|
| | **Parameter** | **R** | **P value** | **F** | **P value** | **$R^2$** | **P value** |
| Technical factors | Paper | 0.962 | 0.001 | 11.486 | 0.001 | 0.537 | 0.001 |
| | Primer | 0.693 | 0.001 | 9.544 | 0.001 | 0.396 | 0.001 |
| | Hypervariable region | 0.655 | 0.001 | 10.653 | 0.001 | 0.339 | 0.001 |
| | Sequencer | 0.506 | 0.001 | 12.540 | 0.001 | 0.190 | 0.001 |
| | Country | 0.356 | 0.001 | 6.481 | 0.001 | 0.238 | 0.001 |
| Biological factors | Lifestyle | 0.439 | 0.001 | 7.608 | 0.001 | 0.125 | 0.001 |
| | Organ | 0.434 | 0.001 | 7.829 | 0.001 | 0.273 | 0.001 |
| | Developmental stage | 0.318 | 0.001 | 6.039 | 0.001 | 0.146 | 0.001 |
