# Supplementary material for: A meta-analysis reveals the environmental and host factors shaping the structure and function of the shrimp microbiota
Source: PeerJ. 2018 Aug 10;6:e5382. doi: 10.7717/peerj.5382 (PMC6089209; doi:10.7717/peerj.5382)
Supplement: Supplemental Information 2 — The rationale for conducting this meta-analysis. [file peerj-06-5382-s018.pdf]

1. **The rationale for conducting this meta-analysis.**

The study of the microbiota in different organisms have changed the view of the impact that the interaction between bacteria, arches, and viruses have on their host's development and health status. In particular, the shrimp is the most valuable traded marine product in the world today. Thus, there are increasing interest in understanding how its microbiota is influenced by external (i.e., habitat, diet) and internal (i.e., age, species) factors.

With the dropping costs of high-throughput sequencing, the studies of shrimp microbiota through 16S rRNA profiling have increased considerably in the last decade. However, due to the different experimental and bioinformatics protocols available, it is difficult to compare and contrast all the available results. Thus, this study aimed to integrate the different studies regarding shrimp microbiota using a consistent set of bioinformatics methods to avoid the technical and bioinformatics differences. We integrated a large number of sequenced samples from all publicly available studies to try to determine the factors that drive and shape the microbiota in this non-model animal.

2. **The contribution that the meta-analysis makes to knowledge in light of previously published related reports, including other meta-analyses and systematic reviews.**

The present study reported the most comprehensive and complete analysis of the microbiota in freshwater and marine shrimps enhancing our understanding of community assembly in this organism independently of the technical and experimental biases introduced by each study. To our knowledge, this is the first systematic meta-analysis that aims to integrate data on 16S shrimp microbiota.

Overall, this meta-analysis establishes how host factors such as age (adult, pre-adult or larva), shrimp organ, shrimp species or health status and external factors such as habitat (marine or freshwater; wt, farm or laboratory). Also, feeding diet can drive the structure of shrimp microbiota setting the basis for future studies that look to analyze the impact of these variables in a particular condition. A meta-analysis like this one can also help to establish the best set of experimental and bioinformatics protocols to characterize the structure and dynamics of the shrimp microbiota.
